# Indole Diterpenoids and Isocoumarin from the Fungus, *Aspergillus flavus*, Isolated from the Prawn, *Penaeus vannamei*

**DOI:** 10.3390/md12073970

**Published:** 2014-06-30

**Authors:** Kunlai Sun, Ye Li, Lei Guo, Yi Wang, Peipei Liu, Weiming Zhu

**Affiliations:** 1 Key Laboratory of Marine Drugs, Ministry of Education of China, School of Medicine and Pharmacy, Ocean University of China, Qingdao 266003, China; E-Mails: sunqinlai@126.com (K.S.); vladimirlee2@gmail.com (Y.L.); wangyi0213@ouc.edu.cn (Y.W.); liupeipei@ouc.edu.cn (P.L.); 2 Key Lab of Marine Biotechnology, Huaihai Institute of Technology, Lianyungang 222005, China; E-Mail: leiguoo@sina.com

**Keywords:** *Penaeus vannamei*, endophytic fungus *Aspergillus flavus*, indole-diterpenoids, bioactivity

## Abstract

Two new indole-diterpenoids (**1** and **2**) and a new isocoumarin (**3**), along with the known β-aflatrem (**4**), paspalinine (**5**), leporin B (**6**), α-cyclopiazonic acid (**7**), iso-α-cyclopiazonic acid (**8**), ditryptophenaline (**9**), aflatoxin B_1_ (**10**), 7-*O*-acetylkojic acid (**11**) and kojic acid (**12**), were isolated from the fermentation broth of the marine-derived fungus, *Aspergillus flavus* OUCMDZ-2205. The structures of Compounds **1**–**12** were elucidated by spectroscopic analyses, quantum ECD calculations and the chemical method. New Compound **1** exhibited antibacterial activity against *Staphylococcus aureus* with a MIC value of 20.5 μM. Both new Compounds **1** and **2** could arrest the A549 cell cycle in the S phase at a concentration of 10 μM. Compound **1** showed PKC-beta inhibition with an IC_50_ value of 15.6 μM. In addition, the absolute configurations of the known compounds, **4**–**6** and leporin A (**6a**), were also determined for the first time.

## 1. Introduction

**Figure 1:**
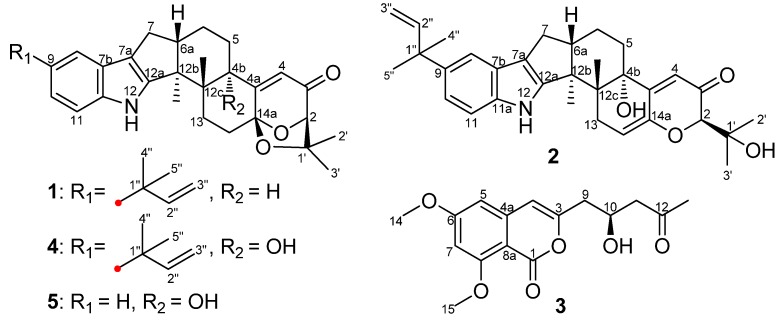
Structures of Compounds **1**–**5** from *Aspergillus flavus* OUCMDZ-2205.

Indole diterpenoids have attracted extensive attention for their diverse skeletons [1,2,3], as well as their different biological activities, such as cytotoxicity [4], antiinsectan activity [1] and tremorgenic activity [5]. Our previous study identified six new and five known indole diterpenoids with anti-H1N1 activity from an aciduric mangrove fungus, *Penicillium camemberti* OUCMDZ-1492 [6]. To enrich the chemodiversity of indole diterpenoids from marine-derived fungi, a fungal strain, *Aspergillus flavus* OUCMDZ-2205, was isolated from the marine prawn, *Penaeus vannamei*. Chemical study resulted in the isolation and identification of two new indole diterpenoids, (2*R*,4b*R*,6a*S*,12b*S*,12c*S*,14a*S*)-4b-deoxy-β-aflatrem (**1**) and (2*R*,4b*S*,6a*S*,12b*S*,12c*R*)-9-isopentenyl paxilline (**2**), and a new isocoumarin, (*S*)-(−)-6,8-di-*O*-methylcitreoisocoumarin (**3**), along with the known β-aflatrem (**4**) [7], paspalinine (**5**) [8], leporin B (**6**) [9], α-cyclopiazonic acid (**7**) [10], iso-α-cyclopiazonic acid (**8**) [11], ditryptophenaline (**9**) [12], aflatoxin B_1_ (**10**) [13], 7-*O*-acetylkojic acid (**11**) [14] and kojic acid (**12**) [15] (Figure 1, Supplementary Figure S2). Compound **1** exhibited antibacterial activity against *Staphylococcus aureus* with a MIC value of 20.5 μM. Both new Compounds **1** and **2** could arrest the A549 cell cycle in the S phase at a concentration of 10 μM. Additionally, Compound **1** showed PKC-beta inhibition with an IC_50_ value of 15.6 μM. Details of the isolation, structure determination and biological activities are presented here.

## 2. Results and Discussion

### 2.1. Structure Elucidation

The EtOAc extract of the fermentation broth of *A*. *flavus* OUCMDZ-2205 was separated by silica gel column, Sephadex LH-20 column and semi-preparative HPLC to give Compounds **1**–**12**.

**Figure 2:**
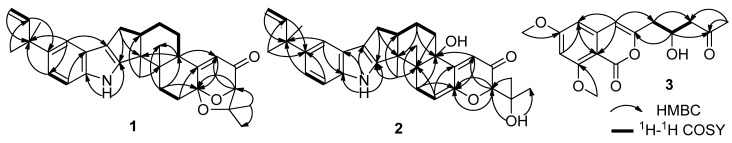
Selected ^1^H-^1^H COSY and HMBC correlations for **1**–**3**.

Compound **1** was obtained as a white amorphous powder. The molecular formula was determined to be C_32_H_39_NO_3_ on the basis of a HRESIMS peak at *m*/*z* 486.2996 [M + H]^+^ (calcd. 486.3008), indicating 14 degrees of unsaturation. The UV spectrum showed characteristic peaks of an indole chromophore at λ_max_ (logε) 235 (4.11) and 245 (4.36) nm [1]. The IR absorption band at 1725 cm^−1^ suggested the presence of a carbonyl group. The ^13^C NMR spectrum was similar to that of β-aflatrem (**4**), except that a methine (δ_C/H_ 40.5/3.12) replaced an oxygenated quaternary carbon (δ_C_ 76.5) (Supplementary Table S1), indicating **1** as an isopentenylated indole diterpenoid. In addition, obvious shifts for C-12c, C-4a and C-5 were observed, suggesting **1** as the deoxy derivative of **4** at C-4b. The deduction was further confirmed by the key COSY correlations of H-4b/H-5/H-6 and the key HMBC correlations from H-4b (δ_H_ 3.12) to C-12b (δ_C_ 51.2), C-4 (δ_C_ 118.7) and Me-12c (δ_C_ 21.6) (Figure 2).

**Figure 3:**
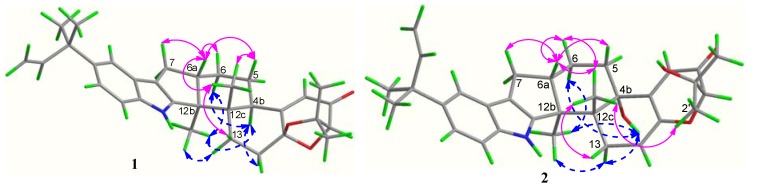
Key NOESY correlations of **1** and **2**.

**Figure 4:**
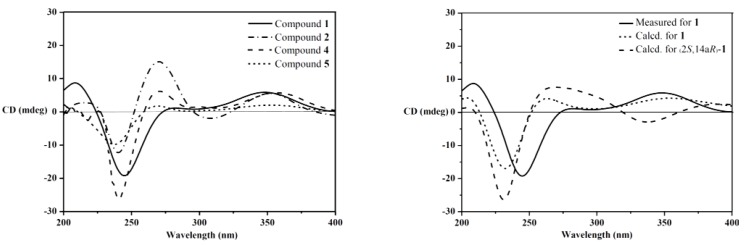
CD spectra of **1**, **2**, **4** and **5** (**left**); measured CD and calculated ECD spectra for **1** and (2*S*,14a*R*)-**1** (**right**).

The relative configuration of **1** was assigned on the basis of the NOESY spectrum. The key NOESY correlations of Me-12b (δ_H_ 1.06) to H-13*α* (δ_H_ 2.40) and H-4b (δ_H_ 3.12) and of Me-12c (δ_H_ 1.03) to H-6a (δ_H_ 2.67) and H-13β (δ_H_ 1.92) (Figure 3) indicated that Me-12b, H-13*α* and H-4b are in the same orientation, while Me-12c, H-13β and H-6a are in the opposite orientation. Furthermore, the same relative configurations of C-14a and C-2 as those of Compounds **4**/**5** could be deduced from the good agreement of ^13^C NMR data of C-2/C-14/C-14a and C-1′/C-2′/C-3′ in **1** and **4**/**5** (Supplementary Table S1). This deduction was confirmed by the quantum chemical calculations of ^13^C NMR for **1** and (2*S*,14a*R*)-**1** at the B3LYP/6-311++G(2d,p)//B3LYP/6-31G(d) level in Gaussian 03 (see Supplementary Information) ([16,17,18,19]). The magnetic shielding values were converted into chemical shifts after the corrections using the slope and intercept of the linear-square functions, and the relative errors of chemical shifts were computed by subtracting the calculated ^13^C NMR from the measured shifts. The maximum error of **1** is less than 13.0 ppm (C-4a), while the maximum error at C-2/C-3/C-1′ reached 23.0/23.1/‒20.5 ppm in (2*S*,14a*R*)-**1**, respectively (Supplementary Table S2). When the configuration changed from (2*R*,14a*S*) to (2*S*,14a*R*), the steric hindrance of Me-12c to C-1′ disappeared, while the steric hindrances of Me-12c to C-2 and Me-2′ to C-3 increased, resulting in the downfield shift of C-1′ and upfield shifts of C-2 and C-3 in (2*S*,14a*R*)-**1**. Thus, the structure of **1** is more reasonable than the fictitious (2*S*,14a*R*)-**1**, further indicating the same relative configurations of **1** and **4**/**5**. In addition, our previous study revealed that the strongest negative CD Cotton effect at a short wavelength (λ_max_ 210–250 nm) arose from the π–π* transitions of the indole nucleus, which could be used to determine the absolute configurations of C-4b, C-6a, C-12b and C-12c of the hexacyclic indole diterpenoids [6]. Thus, the strong negative Cotton effect at λ_max_ 245 (Δ*ε* −8.0) nm (Figure 4) indicated the (4b*R*,6a*S*,12b*S*,12c*S*)-configuration of **1**. The (2*R*,14a*S*)-configuration of **1** could be further supported by the electronic circular dichroism (ECD) calculations for **1** and (2*S*,14a*R*)-**1** using time-dependent density functional theory (TDDFT) [19,20,21,22] at the B3LYP/6-31G(d) level in Gaussian 03. The result showed that the CD curve of **1** is consistent with the calculated ECD curve of **1**, but opposite to that of (2*S*,14a*R*)-**1** at a long wavelength (Figure 4). Thus, the structure of **1** was clearly elucidated as (2*R*,4b*R*,6a*S*,12b*S*,12c*S*,14a*S*)-4b-deoxy-β-aflatrem.

Compound **2** was obtained as a yellowish amorphous powder. The molecular formula was C_32_H_39_NO_4_ on the basis of a HRESIMS peak at *m*/*z* 502.2939 [M + H]^+^ (calcd. 502.2957), indicating 14 degrees of unsaturation. The UV spectrum showed characteristic peaks of an indole diterpenoid nucleus at *λ*_max_ (log*ε*): 233 (4.15) and 248 (4.28) nm. The similarity of ^1^H and ^13^C NMR data between **2** and **4** suggested that Compound **2** is also a hexacyclic indole diterpenoid skeleton. The detailed comparison of NMR data between **2** and **4** revealed that a trisubstituted ethylene moiety (δ_C/H_ 111.4/5.64, 145.6) replaced the corresponding 1,1,1,2-tetrasubstituted ethane moiety (δ_C/H_ 28.4/2.72 & 1.87, 104.7); meanwhile, an additional exchangeable proton (δ_H_ 4.55) emerged in **2**. This result was further confirmed by the key COSY correlations of H_2_-13 (δ_H_ 3.01, 2.40) to H-14 (δ_H_ 5.64) and the key HMBC correlations from H-14 (δ_H_ 5.64) to C-4a (δ_C_ 155.0) and C-12c (δ_C_ 43.3), from H-2 (δ_H_ 4.03) and H-4 (δ_H_ 5.81) to C-14a (δ_C_ 145.6) and from OH-1′ (δ_H_ 4.55) to C-2/1′/2′ (δ_C_ 86.4/73.4/27.6) (Figure 2). Thus, the planar structure of **2** was identified as 9-isopentenylpaxilline. The relative configuration of **2** was assigned on the basis of NOESY spectrum. The NOESY spectrum showed key correlations of Me-12b (δ_H_ 1.29) to H-13α (δ_H_ 3.01) and OH-4b (δ_H_ 4.91) and Me-12c (δ_H_ 1.00) to H-6a (δ_H_ 2.71) and H-13β (δ_H_ 2.40), H_3_-2′ (δ_H_ 1.25) and H_3_-3′ (δ_H_ 1.14) (Figure 3), indicating that Me-12b/H-13α/OH-4b are in the same orientation, while H-13β/H-6a/Me-12c/H_3_-2′ are all in the opposite orientation. The negative CD Cotton effect at λ_max_ 240 (Δε −5.2) nm indicated the (4b*S*,6a*S*,12b*S*,12c*R*)-configuration [6]. Additionally, the (2*R*)-configuration was deduced from the *cis*-orientation of Me-12c and Me-2′, which was further supported by ECD calculation. The ECD calculation revealed that the CD curve of **2** at a long wavelength (λ_max_ 327 nm) is consistent with the calculated ECD curve of **2**, but opposite to its 2-epimer, (2*S*)-**2** (Supplementary Figure S3). Therefore, Compound **2** was accurately identifiedas (2*R*,4b*S*,6a*S*,12b*S*,12c*R*)-9-isopentenylpaxilline.

Although the relative configurations of Compounds **4** [7] and **5** [8] have been published, the absolute configurations have not been firmly established yet. Thus, the same relative configurations of **4** and **5** were established by the agreement of ^1^H and ^13^C NMR data (Supplementary Table S1) with those reported [7,8]. The similar CD Cotton effects of **4** (λ_max_ 240 (Δ*ε* −11.3) and 357 (Δ*ε* +2.6) nm) and **5** (λ_max_ 238 (Δ*ε* −4.2) and 355 (Δ*ε* +0.9) nm) and **1** (λ_max_ 245 (Δ*ε* −8.0), 347 (Δ*ε* +2.5) nm) (Figure 4) indicated that Compounds **4** and **5** shared the same absolute configurations, that is (2*R*,4b*S*,6a*S*,12b*S*,12c*R*,14a*S*). 

The CD curves of Compounds **1**, **4** and **5**, along with our previously reported 2′-hydroxypaxilline, 9,10-diisopentenylpaxilline, paxilline, 21-isopentenylpaxilline and dehydroxypaxilline [6], revealed that all compounds with the (2*R*)-configuration showed a positive CD Cotton effect at λ_max_ 300–350 nm arising from the n–π* transitions of α,β-unsaturated ketone. These data combined with the negative Cotton effects of the calculated ECD of (2*S*,14a*R*)-**1** and (2*S*)-**2** at λ_max_ 300−350 nm revealed that the CD Cotton effects at a long wavelength (λ_max_ 300−360 nm) could be used to determine the absolute configurations of C-2 in the α,β*-*unsaturated lactone moiety of hexacyclic indole diterpenoids. The positive effect means the (2*R*)-configuration, while the negative effect means (2*S*)-configuration.

**Figure 5:**
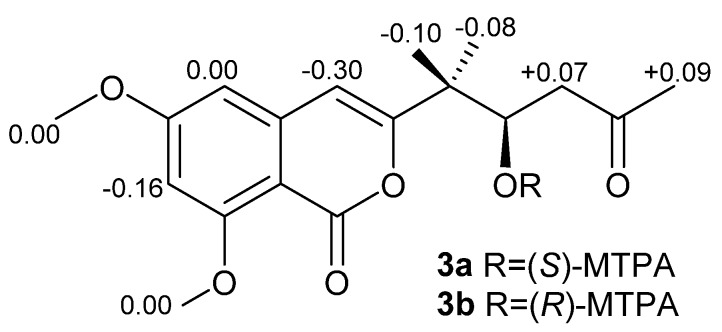
Δδ (δ*_S_*-δ*_R_*) values of the two MTPA esters of **3**.

Compound **3** was obtained as a light yellow solid. The molecular formula was established as C_16_H_18_O_6_ by an HRESIMS peak at *m*/*z* 307.1180 [M + H]^+^ (calcd. 307.1176). The UV spectrum at λ_max_ (logε) 236 (3.85) and 245 (4.18) nm indicated an isocoumarin nucleus [23]. Its ^1^H and ^13^C NMR data were similar to those of citreoisocoumarin with the exception of two additional methoxy groups at δ_C/H_ 56.3/3.87 and 56.5/3.86 [24,25], indicating **3** as the derivative of citreoisocoumarin. The key HMBC correlations from H-14 (δ_H_ 3.87) to C-6 (δ_C_ 165.6) and from H-15 (δ_H_ 3.86) to C-8 (δ_C_ 163.2) (Figure 2) supported that Compound **3** is the 6,8-di-*O*-methyl derivative of citreoisocoumarin. The *S*-configuration of **3** was deduced from the same specific rotation as that of (*S*)-(−)-citreoisocoumarin ([α]_D_ −10) [24]. This deduction was further confirmed from the result of the Mosher’s method [26]. The distribution of Δδ values between (*S*)-and (*R*)-MTPA (α-methoxy-α-(trifluoromethyl)phenylacetic acid) esters (**3a** and **3b**) clearly indicated the (*S*)-configuration (Figure 5 and Supplementary Information). Thus, Compound **3** was unambiguously determined as (*S*)-(−)-6,8-di-*O*-methyl citreoisocoumarin.

The ^1^H and ^13^C NMR data and [α]_D_ value of Compound **6** were in agreement with those reported for leporin B [9] (see Supplementary Information). Since there were no reports on the absolute configurations of leporin B (**6**) and its related leporin A (**6a**) in the literature, the CD spectrum was measured and the ECD was calculated. The results showed that the CD curve of **6** was in good agreement with the calculated ECD of the structure of **6**, but opposite of the calculated ECD of *ent*-**6** (Supplementary Figure S4). Thus, the absolute configuration of leporin B (**6**) was determined as (7*S*,8*R*,12*R*,13*S*). To determine the absolute configuration of leporin A (**6a**), Compound **6a** was prepared from the methylation of **6** (see Supplementary Information). The synthetic-**6a** displayed the same ^1^H and ^13^C NMR data and a similar specific rotation as those reported (−36 *vs.* −22) [27]. Thus, the absolute configuration of leporin A (**6a**) is also (7*S*,8*R*,12*R*,13*S*).

Indole diterpenoids isolated from fungus can be traced back to 1980, and by now, 49 new indole diterpenoids have been identified from the fungal metabolites. According to the literature tracking, indole diterpenoids were produced by *Aspergillus* sp*.* [4], *Penicillium* sp*.* [6], *Eupenicillium* sp. [28], *Dichotomomyces* sp. [29], *Claviceps* sp. [30] and *Nodulisporium* sp*.* [31], respectively. This situation is common in the microbial community, that different microbes could produce the same type of natural products. We speculate that these different genera and species of fungi might have partial, similar biosynthetic gene clusters, which might be the reason why the same type of natural products can be isolated from different fungal metabolites.

### 2.2. Bioactivities the of Compounds Produced by Aspergillus flavus OUCMDZ-2205

Compounds **1**–**10** were assayed for their cytotoxic effects on the MCF-7 and A549 cell line by the MTT method [32] and the antibacterial activity against *Enterobacter aerogenes*, *Bacillus subtilis*, *Escherichia coli*, *Pseudomonas aeruginosa*, *Staphylococcus aureus* and *Candida albicans* using the agar dilution method [33], respectively. In addition, new Compounds **1** and **2** were also evaluated for their inhibitory effects on the A549 cell cycle [34] and the kinase, PKC-beta [35]. Because of a lack of quantity, only Compounds **4** and **6**–**12** were evaluated for their antiviral activities against the H1N1 virus by the CPE (cytopathic effect) inhibition assay [36]. The results showed that Compound **1** exhibited antibacterial activity against *Staphylococcus aureus* with a MIC value of 20.5 μM. The indole diterpenes (**1**, **2**, **4** and **5**) displayed weak cytotoxicity against MCF-7 and A549 cells with IC_50_ values of 18‒30 μM (Supplementary Table S3). Additionally, Compounds **1** and **2** could arrest the A549 cell cycle in the S phase at a concentration of 10 μM (Supplementary Figure S24), and **1** showed PKC-beta inhibition with an IC_50_ value of 15.6 μM (IC_50_ > 100 μM for **2**). No anti-H1N1 activity (IC_50_ > 150 μM) was observed for Compounds **4** and **6**–**12**, indicating that the further oxidation at C-14a decreases the anti-H1N1 activity of hexacyclic indole diterpenoids [6]. PKC beta mediates the signaling of inflammatory, mitogenic and angiogenic effects [37] and participates in the occurrence and development of colon cancer, breast cancer, lung cancer and neuroblastoma [38]. PKC-beta inhibitors are a new class of drugs that are effective in attenuating the vascular complications of diabetes [37] and had turned out to be a new ideal research target for anticancer drugs [38]. The weak cytotoxicity, the cell cycle arresting and the PKC-beta inhibition of **1** indicated that it could be a potential target compound for anticancer drug discovery.

## 3. Experimental Section

### 3.1. General Experimental Procedures

Optical rotations were measured with a JASCO P-1020 digital polarimeter (JASCO Corporation, Tokyo, Japan). UV spectra were recorded on a Waters 2487 absorbance detector (Waters Corporation, Milford, MA, USA). CD spectra were measured on a JASCO J-715 and a J-815 spectropolarimeter (JASCO Corporation, Tokyo, Japan). IR spectra were taken on a Nicolet NEXUS 470 spectrophotometer (Nicolet Instrument Corporation, Madison, WI, USA) as KBr disks. ^1^H and ^13^C NMR, DEPT and 2D NMR spectra were recorded on a JEOL JNM-ECP 600 (JEOL Ltd, Tokyo, Japan) for Compounds **1**−**12** using TMS as the internal standard, and chemical shifts were recorded as δ values. ESIMS was measured on a Q-TOF ULTIMA GLOBAL GAA076 LC mass spectrometer (Micromass UK Ltd., Manchester, UK). Semi-preparative HPLC was performed using an ODS column (YMC-pack ODS-A, 10 × 250 mm, 5 μm, 4 mL/min). TLC and column chromatography (CC) were performed on plates precoated with silica gel GF_254_ (10−40 μm) and over silica gel (200–300 mesh, Qingdao Marine Chemical Factory, Qingdao, China) and Sephadex LH-20 (Amersham Biosciences, Uppsala, Sweden). Vacuum-liquid chromatography (VLC) was carried out over silica gel H (Qingdao Marine Chemical Factory, Qingdao, China). Sea salt was made by the evaporation of seawater collected in Laizhou Bay (Weifang Haisheng Chemical Factory, Weifang, China).

### 3.2. Fungal Material

The fungal strain, *Aspergillus flavus* OUCMDZ-2205, was isolated from the prawn, *Penaeus vannamei*, from the Lianyungang sea area, Jiangsu Province of China, in 2012. The prawn sample was successively washed with 75% EtOH and sterile seawater. Then, the sample was mashed with a mortar and diluted to 10^−2^ with sterile water. The 100 µL of diluents were evenly coated on potato dextrose agar medium (PDA; per liter containing potato extract, 200 g, glucose, 20 g, agar ,15 g, and seawater, 1 L) and cultivated at 28 °C for 7 days. The colony was collected and purified. It was identified according to its morphological characteristics and 18S rRNA gene sequence (GenBank Accession No. KC120773). A voucher specimen was deposited in Zhu’s laboratory at −80 °C. The working strain was prepared on PDA slants and stored at 4 °C.

### 3.3. Fermentation and Extraction

The fungus, *A. flavus* OUCMDZ-2205, was cultured in 1 L-conical flask containing 300 mL fermentation medium that was composed of glucose (10 g/L), maltose (20 g/L), mannitol (20 g/L), monosodium glutamate (10 g/L), KH_2_PO_4_ (0.5 g/L), MgSO_4_·7H_2_O (0.3 g/L), yeast extract (3 g/L), sea salt (33 g/L) and tap water (1 L, pH 7.0) and was grown under static conditions for 32 days at 25 °C. The fermented whole broth (25 L) was filtered through cheesecloth to separate the filtrate from the mycelia. Then, the filtrate was extracted three times with an equivalent volume of EtOAc to give an EtOAc solution. The mycelia were extracted three times with 80% acetone. The acetone solution was concentrated under reduced pressure to afford an aqueous solution that was extracted three times with an equivalent volume of EtOAc to give another EtOAc solution. Both EtOAc solutions were combined and concentrated under reduced pressure to give EtOAc extract (70.2 g).

### 3.4. Purification and Identification

The EtOAc extract (70.2 g) was subjected to a silica gel VLC column, eluting with a stepwise gradient of petroleum ether–CH_2_Cl_2_ (1:1 and 0:1) and then of CH_2_Cl_2_–MeOH (100%–0%) to yield eight primary fractions (Fractions 1–8). Fraction 2 (3.8 g) eluted with petroleum CH_2_Cl_2_–MeOH (100:1) was further purified by Sephadex LH-20 (1:1 CH_2_Cl_2_–MeOH) to afford Fraction 2.1 and Fraction 2.2, which were purified by semi-preparative HPLC eluting with 90% MeOH–H_2_O to yield Compounds **1**(2.0 mg, *t*_R_6.0 min) and **2** (2.3 mg, *t*_R_8.0 min), respectively. Fraction 3 (7.7 g) eluted with CH_2_Cl_2_–MeOH (90:1) was subjected to RP-18 silica column eluting with a stepwise gradient of 40%–100% MeOH/H_2_O to give four subfractions (Fractions 3.1–3.4). Fraction 3.1 (2.4 g) was further purified by Sephadex LH-20 eluting with CH_2_Cl_2_–MeOH (1:1) to give Fractions 3.1.1–3.1.3. Fraction 3.1.3 (180 mg) was purified by semi-preparative HPLC eluting with 85% MeOH/H_2_O to yield Compounds **5** (1.3 mg, *t*_R_ 16.5 min) and **3** (3.0 mg, *t*_R_ 10.0 min). Fraction 3.3 (800 mg) was subjected to a Sephadex LH-20 column eluting with CH_2_Cl_2_–MeOH (1:1) and further purified by semi-preparative HPLC (85% MeOH/H_2_O) to yield **6** (6.4 mg, *t*_R_ 17.8 min). Similarly, Fraction 3.4 (1.2 g) was subjected to a Sephadex LH-20 column (1:1 CH_2_Cl_2_–MeOH) and then was further purified by semi-preparative HPLC (80% MeOH/H_2_O) to yield Compounds **7** (3.3 mg, *t*_R_ 7.3 min) and **12** (4.1 mg, *t*_R_ 10.6 min). Fraction 4 (6.1 g) eluted with CH_2_Cl_2_–MeOH (80:1) was further subjected to RP-18 silica column eluting with a stepwise gradient of 30%–100% MeOH/H_2_O to afford four subfractions (Fractions 4.1–4.4). Fraction 4.1 (2.0 g) was further isolated by Sephadex LH-20 eluting with MeOH to provide Fraction 4.1.1 and Fraction 4.1.2. Compounds **10** (5 mg, *t*_R_ 7.3 min) and **11** (6.4 mg, *t*_R_ 3.9 min) were purified from Fraction 4.1.1 and Fraction 4.1.2 by semi-preparative HPLC eluting with 75% MeOH/H_2_O, respectively. Furthermore, Fraction 4.3 (130 mg) was further purified by Sephadex LH-20 with MeOH and then was purified by semi-preparative HPLC (70% MeOH/H_2_O) to yield **8** (3.2 mg, *t*_R_ 10.0 min). Fraction 4.4 (1.6 g) was subjected to Sephadex LH-20 column (MeOH) to give Fraction 4.4.1 and Fraction 4.4.2. Fraction 4.4.1 (96 mg) and Fraction 4.4.2 (85 mg) were further purified by semi-preparative HPLC eluting with 70% MeOH/H_2_O and 80% MeOH/H_2_O to afford Compounds **9** (6.1 mg, *t*_R_ 6.7 min) and **4** (4.8 mg, *t*_R_ 16.3 min), respectively.

(2*R*,4b*R*,6a*S*,12b*S*,12c*S*,14a*S*)-4b-Deoxy-β-aflatrem (**1**): white amorphous powder; [α]_D_^20^ +62 (*c* 2.2, CHCl_3_); UV (MeOH) λ_max_ (logε) 235 (4.11), 245 (4.36) nm; CD (*c* 0.1, MeOH) λ_max_ (Δε) 209 (+3.8), 245 (−8.0), 280 (+0.5), 347 (+2.5) nm; IR (KBr) ν_max_ 3396, 3334, 2922, 2851, 1664, 1606, 1446 cm^−1^; ^1^H and ^13^C NMR data; see Supplementary Table S1; HRESIMS *m*/*z* 486.2996 [M + H]^+^ (calcd. for C_32_H_40_NO_3_, 486.3008).

(2*R*,4b*S*,6a*S*,12b*S*,12c*R*)-9-Isopentenylpaxilline D (**2**): light yellow amorphous powder; [α]_D_^20^ +17 (*c* 0.4, CHCl_3_); UV (MeOH) λ_max_ (logε) 233 (4.15), 248 (4.28) nm; CD (*c* 0.3, MeOH) λ_max_ (Δε): 240 (−5.2), 270 (+6.5), 307 (−0.9), 350 (+2.5) nm; IR (KBr) ν_max_ 3396, 3334, 2922, 2851, 1664, 1606, 1446; ^1^H and ^13^C NMR data; see Supplementary Table S1; HRESIMS *m*/*z* 502.2939 [M + H]^+^ (calcd. for C_32_H_40_NO_4_, 502.2957).

(*S*)-(−)-6,8-Di-*O*-Methylcitreoisocoumarin (**3**): light yellow solid; [α]_D_^20^ −10 (*c* 2.0, CHCl_3_); UV (MeOH) λ_max_ (logε) 209 (3.38), 236 (3.85), 245 (4.18); ^1^H NMR (600 MHz, DMSO-*d*_6_) δ 6.38 (s, 1H, H-4), 6.61 (d, 1H, *J* = 2.2 Hz, H-5), 6.57 (d, 1H, *J* = 2.3 Hz, H-7), 2.48 (dd, 1H, *J* = 14.3, 7.9 Hz, H-9a), 2.54 (dd, 1H, *J* = 14.5, 5.1 Hz, H-9b), 4.27 (m, 1H, H-10), 2.57 (d, 2H, *J* = 6.2 Hz, H-11), 2.11 (s, 3H, H-13), 3.87 (s, 3H, H-14), 3.86 (s, 3H, H-15), 5.06 (s, 1H, HO-10); ^13^C NMR (150 MHz, DMSO-*d*_6_) δ 158.5 (C, C-1), 156.1 (C, C-3), 105.2 (CH, C-4), 142.3 (C, C-4a), 100.8 (CH, C-5), 165.6 (C, C-6), 98.8 (CH, C-7), 163.2 (C, C-8), 102.4 (C, C-8a), 41.4 (CH_2_, C-9), 65.0 (CH, C-10), 50.8 (CH_2_, C-11), 207.9 (C, C-12), 31.0 (CH_3_, C-13), 56.3 (CH_3_, C-14), 56.5 (CH_3_, C-15); HRESIMS *m*/*z* 307.1180 [M + H]^+^ (calcd. for C_16_H_19_O_6_, 307.1176).

### 3.5. Supplementary Information Available

Following information can be found in supplementary file: 18S rRNA gene sequences of *A*. *flavus* OUCMDZ-2205; bioassay protocols used; the physicochemical data of the known compounds, **4**–**12** and **6a**; chemical transformation of leporin B (**6**) into leporin A (**6a**); X-ray crystal data for **9** and **12**; theory and calculation details; ^13^C NMR quantum chemical calculations of **1** and (2*S*,14a*R*)-**1**; crystal structures of **9** and **12**; measured CD and calculated ECD spectra of **2** and **6**; the NMR spectra of Compounds **1**–**3**; the cytotoxicities of Compounds **1**–**10**; and the preparation of MTPA esters **3a** and **3b** and their ^1^H NMR data.

## 4. Conclusions

Three new compounds, (2*R*,4b*R*,6a*S*,12b*S*,12c*S*,14a*S*)-4b-deoxy-β-aflatrem (**1**), (2*R*,4b*S*,6a*S*,12b*S*, 12c*R*)-9-isopentenylpaxilline D (**2**) and (*S*)-(−)-6,8-Di-*O*-Methylcitreoisocoumarin (**3**), have been identified from the fermentation broth of the marine-derived *A*. *flavus* OUCMDZ-2205. Compound **1** exhibited anti-*S*. *aureus* and PKC-beta inhibition with MIC and IC_50_ values of 20.5 and 15.6 μM, respectively. Both **1** and **2** could arrest the A549 cell cycle at the S phase at a concentration of 10 μM. In addition, the absolute configurations of the known compounds **4**–**6** and leporin A (**6a**) were also determined for the first time. 

## Supplementary Files

Supplementary File 1 Supplementary Information (PDF, 2206 KB)
