# Supplementary material for: Indole Diterpenoids and Isocoumarin from the Fungus, Aspergillus flavus, Isolated from the Prawn, Penaeus vannamei
Source: Mar Drugs. 2014 Jun 30;12(7):3970–81. doi: 10.3390/md12073970 (PMC4113809; doi:10.3390/md12073970)
Supplement: Supplementary File 1 — Supplementary Information (PDF, 2206 KB) [file marinedrugs-12-03970-s001.pdf]

Supplementary Information

S1. 18S rRNA Gene Sequences of *Aspergillus flavus* OUCMDZ-2205

GCACTTTATACTGTGAAACTGCGAATGGCTCATTAAATCAGTTATCGTTTATTTGATAGTACC
TTACTACATGGATACCTGTGGTAATTCTAGAGCTAATACATGCTAAAAACCTCGACTTCGGA
AGGGGTGTATTTATTAGATAAAAAACCAATGCCCTTCGGGGCTCCTTGGTGATTCATAATAA
CTTAACGAATCGCATGGCCTTGCGCCGGCGATGGTTCATTCAAATTTCTGCCCTATCAACTT
TCGATGGTAGGATAGTGGCCTACCATGGTGGCAACGGGTAACGGGGAATTAGGGTTCGATT
CCGGAGAGGGAGCCTGAGAAACGGCTACCACATCCAAGGAAGGCAGCAGGCGCGCAAAT
TACCCAATCCCGACACGGGGAGGTAGTGACAATAAATACTGATACGGGGCTCTTTTGGGTC
TCGTAATTGGAATGAGTACAATCTAAATCCCTTAACGAGGAACAATTGGAGGGCAAGTCTG
GTGCCAGCAGCCGCGGTAATTCCAGCTCCAATAGCGTATATTAAAGTTGTTGCAGTTAAAA
AGCTCGTAGTTGAACCTTGGGTCTGGCTGGCCGGTCCGCCTCACCGCGAGTACTGGTCCGG
CTGGACCTTTCCTTCTGGGGAACCTCATGGCCTTCACTGGCTGTGGGGGGAACCAGGACTT
TTACTGTGAAAAAATTAGAGTGTTCAAAGCAGGCCTTTGCTCGAATACATTAGCATGGAATA
ATAGAATAGGACGTGCGGTTCTATTTTGTTGGTTTCTAGGACCGCCGTAATGATTAATAGGG
ATAGTCGGGGGCGTCAGTATTCAGCTGTCAGAGGTGAAATTCTTGGATTTGCTGAAGACTA
ACTACTGCGAAAGCATTCGCCAAGGATGTTTTCATTAATCAGGGAACGAAAGTTAGGGGAT
CGAAGACGATCAGATACCGTCGTAGTCTTAACCATAAACTATGCCGACTAGGGATCGGGCG
GTGTTTCTATGATGACCCGCTCGGCACCTTACGAGAAATCAAAGTTTTTGGGTTCTGGGGG
GAGTATGGTCGCAAGGCTGAAACTTAAAGAAATTGACGGAAGGGCACCACAAGGCGTGG
AGCCTGCGGCTTAATTTGACTCAACACGGGGAAACTCACCAGGTCCAGACAAAATAAGGA
TTGACAGATTGAGAGCTCTTTCTTGATCTTTTGGATGGTGGTGCATGGCCGTTCTTAGTTGG
TGGAGTGATTTGTCTGCTTAATTGCGATAACGAACGAGACCTCGGCCCTTAAATAGCCCGG
TCCGCGTTTGCGGGCCGCTGGCTTCTTAGGGGGACTATCGCTCAAG

Figure S1. Polymeric analysis of OUCMDZ-2205 and other strains based on the 18S rRNA gene sequences.

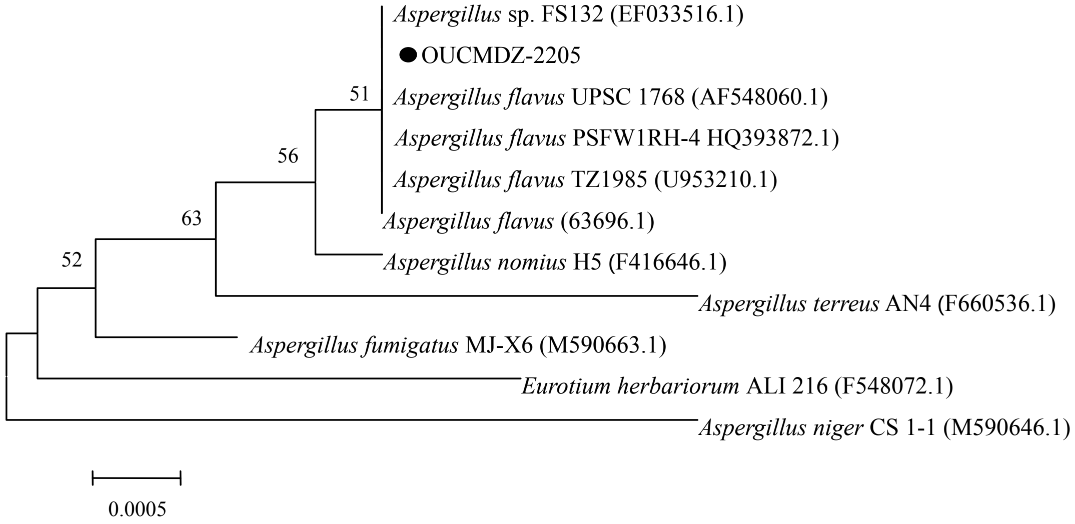

S2. Bioassay Protocols

Cytotoxic Assays. Cytotoxicity activity was assayed by the MTT method [1]. In the MTT assay, the MCF-7 and A549 cell lines were grown in RPMI-1640 supplemented with 10% FBS under a humidified atmosphere of 5% CO_2 and 95% air at 37 °C. The cell suspension, 200 μL, at a density of 5×10^4 cell·mL^{-1}, was plated in 96-well microtiter plates and incubated for 24 h. Then, 2 μL of the test solutions (in DMSO) were added to each well and further incubated for 72 h. The MTT solution (20 μL, 5 mg/mL in IPMI-1640 medium) was then added to each well and incubated for 4 h. Old medium containing MTT (150 μL) was then gently replaced by DMSO and pipetted to dissolve any formazan crystals formed. Absorbance was then determined on a Spectra Max Plus plate reader at 540 nm. Adriamycin was used as a positive control against the MCF-7 and A549 cell lines with IC_{50} values of 0.35 μM and 0.15 μM, respectively.

Antimicrobial Assays. The antimicrobial activities against *Escherichia coli*, *Bacillus aerogenes*, *Pseudomonas aeruginosa*, *Bacillus subtilis*, *Staphylococcus aureus* and *Candida albicans* were evaluated by an agar dilution method [2]. The tested strains were cultivated in LB agar plates for bacteria and in YPD agar plates for *Candida albicans* at 37 °C. Compounds **1**–**10** and positive controls were dissolved in MeOH at different concentrations from 100 to 0.05 μg/mL by the continuous two-fold dilution methods. A 10-μL quantity of test solution was absorbed by a paper disk (5 mm diameter) and placed on the assay plates. After 12 h of incubation, zones of inhibition were observed. The minimum inhibitory concentrations (MIC) were defined as the lowest concentration at which no microbial growth could be observed. Ciprofloxacin lactate and ketoconazole was used as the positive control for *Escherichia coli*, *Bacillus aerogenes*, *Pseudomonas aeruginosa*, *Bacillus subtilis*, *Staphylococcus aureus* and *Candida albicans* with MIC values of 0.24, 0.47, 0.12, 0.94, 3.78 and 0.02 μM, respectively.

Cell Cycle Analysis. A549 cells were treated with DMSO (negative control), Compounds **1** and **2** at 37 °C for 48 h. Then, cells were digested with 0.5 g/L trypsin, collected, washed twice with cold PBS and fixed in 4% paraformaldehyde for 15 min. The cells were stained with 500 μL of hypotonic PI buffer containing 50 μg/mL PI, 0.1 mg/mL RNase A and 0.05% Tritin X-100 for 30 min at 37 °C. The obtained solution was centrifuged at 1500 rpm for 5 min and suspended in PBS. The cell cycle distribution was determined using a BD FACS Calibur Flow Cytometer, and the data were analyzed using CellQuest and ModFit software [3].

Kinase Assay in Vitro [4]. The recombinant kinase PKC-beta was expressed in High Five insect cells as a fusion protein with a 6× His tag at the *C*-terminus. It was purified by immobilized metal chelate affinity chromatography on Ni-NTA beads. All kinase reactions were performed in a final assay volume of 10 μL using the Z-Lyte assay kit (Invitrogen, Carlsbad, CA, USA) and monitoring on an EnVision plate reader. Raw fluorescence values were converted to the concentration of product formed using substrate and product standards. The kinase inhibition test was carried out with these compounds, of which the final concentration was 100 μM, and each sample was tripled in one plate. The IC_{50} data was calculated using the software, GraphPad Prism, and the equation "sigmoidal dose-response (variable slope)" for curve fitting was chosen. Staurosporine was used as the positive control with an IC_{50} value of 0.12 μM.

Anti-Influenza A Virus (H1N1) Bioassay. The antiviral activity against H1N1 was evaluated by the CPE inhibition assay [5]. Confluent MDCK cell monolayers were firstly incubated with influenza virus (A/Puerto Rico/8/34 (H1N1), PR/8) at 37 °C for 1 h. After removing the virus dilution, cells were maintained in infecting media (RPMI 1640, 4 μg/mL of trypsin) containing different concentrations of test compounds at 37 °C. After 48 h of incubation at 37 °C, the cells were fixed with 100 μL of 4% formaldehyde for 20 min at room temperature. After the removal of the formaldehyde, the cells were stained with 0.1% crystal violet for 30 min. The plates were washed and dried, and the intensity of crystal violet staining for each well was measured in a microplate reader (Bio-Rad, Hercules, CA, USA) at 570 nm. The IC_{50} was calculated as the compound concentration required to inhibit the influenza virus yield at 48 h post-infection by 50%. Ribavirin was used as the positive control with an IC_{50} value of 113.1 μM.

Figure S2. Structures of Compounds **6–12** and **6a**.

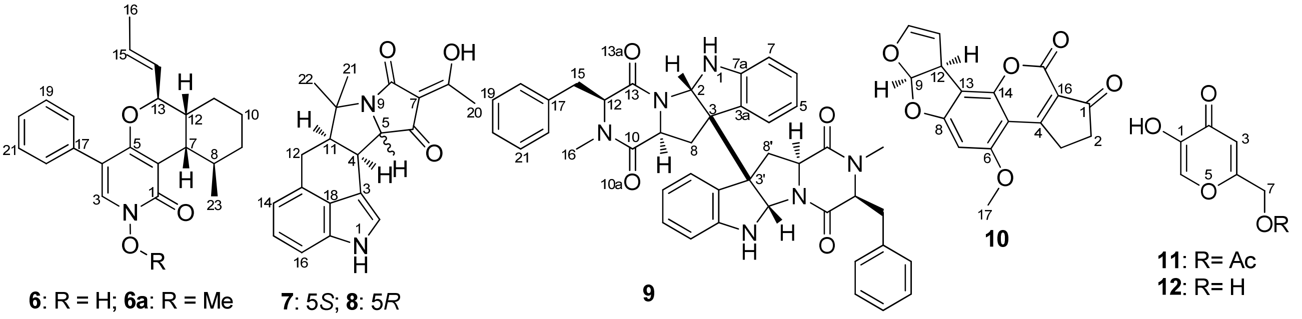

S3. The Physicochemical Data of the Known Compounds

β-Aflatrem (**4**): Yellow amorphous solid; $[\alpha]_D^{20}$ +68 (*c* 1.2, $CHCl_3$); UV (MeOH) λ_{max} (logε) 235 (4.13), 245 (4.31) nm; CD (*c* 0.1, MeOH) λ_{max} (Δε) 240 (−11.3), 271 (+2.4), 305 (+0.8), 357 (+2.4) nm; ^1H and ^{13}C NMR data; see Table S1; ESI-MS *m*/*z* 502.3 $[M + H]^+$.

Paspalinine (**5**): Yellow amorphous solid; $[\alpha]_D^{20}$ +56 (*c* 1.0, $CHCl_3$); UV (MeOH) λ_{max} (logε) 232 (4.10), 246 (4.30) nm; CD (*c* 0.3, MeOH) λ_{max} (Δε) 238 (−4.2), 267 (+0.8), 355 (+0.9) nm; ^1H and ^{13}C NMR data; see Table S1; ESI-MS *m*/*z* 434.3 $[M + H]^+$.

Leporin B (**6**): Yellow oil; $[\alpha]_D^{20}$ −57 (*c* 1.1, $CHCl_3$); UV (MeOH) λ_{max} (logε) 230 (4.05), 235 (4.11), 244 (4.36) nm; CD (*c* 0.1, MeOH) λ_{max} (Δε) 207 (−0.6), 247 (+1.0), 270 (−0.8) nm; ESI-MS *m*/*z* 352.2 $[M + H]^+$; ^1H NMR (600 MHz, $CDCl_3$) δ 7.68 (1H, s, H-3), 7.42 (2H, d, *J* = 7.5 Hz, H-18/22), 7.34 (2H, dd, *J* = 7.4, 7.5 Hz, H-19/21), 7.27 (1H, dd, *J* = 7.3, 7.3 Hz, H-20), 5.79 (1H, dq, *J* = 15.1, 6.4 Hz, H-15), 5.40 (1H, dd, *J* = 15.0, 8.2 Hz, H-14), 4.86 (1H, dd, *J* = 10.5, 8.2 Hz, H-13), 2.67 (1H, dd, *J* = 10.6, 3.3 Hz, H-7), 1.72 (1H, overlap, H-12), 1.69 (2H, overlap, H-11), 1.68 (3H, d, *J* = 6.3 Hz, H-16), 1.67 (1H, overlap, H-8), 1.60 (2H, m, H-9), 1.45 (2H, m, H-10), 0.88 (3H, d, *J* = 6.3 Hz, H-23). ^{13}C NMR (150 MHz, $CDCl_3$) δ 159.0 (C, C-1), 157.8 (C, C-5), 133.7 (C, C-17), 131.1 (CH, C-3), 131.1 (CH, C-15), 129.3 (CH, C-14), 129.2 (2× CH, C-18/22), 128.3 (2× CH, C-19/21), 127.3 (CH, C-20), 113.8 (C, C-6), 111.4 (C, C-4), 78.1 (CH, C-13), 37.9 (CH, C-7), 35.8 (CH, C-12), 35.8 (CH, C-8), 35.1 (CH_2, C-9), 26.4 (CH_2, C-11), 20.8 (CH_3, C-23), 20.3 (CH_2, C-10), 17.8 (CH_3, C-16).

Leporin A (**6a**): Yellow oil; $[\alpha]_D^{20}$ −36 (*c* 1.4, CH_2Cl_2); ESI-MS *m/z* 366.2 $[M + H]^+$; ^{13}C NMR (125 MHz, DMSO-d_6) δ 158.6 (C, C-1), 157.9 (C, C-5), 133.5 (C, C-17), 131.4 (CH, C-3), 131.0 (CH, C-15), 129.3 (CH, C-14), 129.0 (2× CH, C-18/22), 128.2 (2× CH, C-19/21), 127.3 (CH, C-20), 114.1 (C, C-4), 110.0 (C, C-6), 78.1 (CH, C-13), 64.8 (CH_3, OCH_3), 37.7 (CH, C-7), 36.0 (CH, C-8), 35.8 (CH_2, C-9), 35.2 (CH, C-12), 26.4 (CH_2, C-11), 20.8 (CH_2, C-10), 20.6 (CH_3, C-23), 17.8 (CH_3, C-16).

α-Cyclopiazonic acid (**7**): Yellow amorphous solid; $[\alpha]_D^{20}$ −21 (*c* 1.0, $CHCl_3$); UV (MeOH) λ_{max} (logε) 230 (3.40), 282 (4.11) nm; CD (*c* 0.1, MeOH) λ_{max} (Δ*ε*) 210 (+3.3), 245 (−1.5), 305(+2.7) nm; ESI-MS *m/z* 337.1 $[M + H]^+$; ^1H NMR (600 MHz, DMSO-d_6) δ 10.86 (1H, s, H-1), 7.17 (1H, d, *J* = 7.8 Hz, H-14), 7.06 (1H, s, H-2), 7.02 (1H, dd, *J* = 7.6, 6.2 Hz, H-15), 6.80 (1H, d, *J* = 6.0 Hz, H-16), 4.15 (1H, d, *J* = 10.7 Hz, H-5), 3.64 (1H, dd, *J* = 10.9, 5.4 Hz, H-4), 3.04 (1H, dd, *J* = 15.8, 13.2 Hz, H-12β), 2.95 (1H, dd, *J* = 15.8, 4.5 Hz, H-12α), 2.55 (1H, m, H-11), 2.39 (3H, s, H-20), 1.60 (3H, s, H-21), 1.54 (3H, s, H-22). ^{13}C NMR (150 MHz, DMSO-d_6) *δ* 194.8 (C, C-6), 184.1 (C, C-19), 171.8 (C, C-8), 133.7 (C, C-17), 129.4 (C, C-13), 127.6 (C, C-18), 123.0 (CH, C-15), 120.1 (CH, C-2), 115.7 (CH, C-14), 108.8 (CH, C-16), 107.9 (C, C-3), 106.5 (C, C-7), 71.3 (CH, C-5), 62.2 (C, C-10), 52.3 (CH, C-11), 35.5 (CH, C-4), 30.0 (CH_3, C-21), 26.1 (CH_2, C-12), 21.6 (CH_3, C-22), 20.0 (CH_3, C-20).

iso-α-Cyclopiazonic acid (**8**): Yellow amorphous solid; $[\alpha]_D^{20}$ +81 (*c* 1.2, $CHCl_3$); UV (MeOH) λ_{max} (logε) 230 (3.50), 282 (4.21) nm; CD (*c* 0.1, MeOH) λ_{max} (Δε) 208 (−1.4), 243 (+2.2), 274 (−2.6), 313 (+1.0) nm; ESI-MS *m/z* 337.1 $[M + H]^+$; ^1H NMR (600 MHz, DMSO-d_6) δ 10.64 (1H, s, H-1), 7.08 (1H, d, *J* = 7.8 Hz, H-14), 7.00 (1H, dd, *J* = 7.8, 7.0 Hz, H-15), 6.76 (1H, d, *J* = 7.0 Hz, H-16), 6.72 (1H, s, H-2), 4.68 (1H, d, *J* = 5.5 Hz, H-5), 3.75 (1H, overlap, H-4), 3.18 (1H, dd, *J* = 15.0, 6.4 Hz, H-11), 2.95 (2H, overlap, H-12), 2.39 (3H, s, H-20), 1.41 (3H, s, H-21), 0.72 (3H, s, H-22). ^{13}C NMR (150 MHz, DMSO-d_6) δ 195.1 (C, C-6), 184.0 (C, C-19), 175.1 (C, C-8), 133.7 (C, C-17), 129.2 (C, C-13), 126.3 (C, C-18), 122.5 (CH, C-15), 121.7 (CH, C-2), 115.9 (CH, C-14), 109.2 (CH, C-16), 109.2 (C, C-3), 105.9 (C, C-7), 71.6 (CH, C-5), 63.0 (C, C-10), 53.0 (CH, C-11), 36.0 (CH, C-4), 26.4 (CH_3, C-21), 26.2 (CH_2, C-12), 24.7 (CH_3, C-22), 19.3 (CH_3, C-20).

Ditryptophenaline (**9**): White crystal solid; $[\alpha]_D^{20}$ −352 (*c* 0.9, MeOH); UV (MeOH) λ_{max} (logε) 235 (3.93), 238 (4.03), 245 (4.13) nm; CD (*c* 0.1, MeOH) λ_{max} (Δ*ε*) 220 (+4.6), 248 (−13.0), 275 (−2.9), 305 (−10.8) nm; ESI-MS *m/z* 715.3 $[M + Na]^+$; ^1H NMR (600 MHz, DMSO-d_6) symmetrical: *δ* 7.49 (2H, dd, *J* = 7.5, 7.5 Hz, H-19/21), 7.37 (1H, dd, *J* = 7.5, 7.5 Hz, H-20), 7.10 (2H, d, *J* = 7.4 Hz, H-18/22), 7.02 (1H, dd, *J* = 7.5, 7.4 Hz, H-6), 6.98 (1H, d, *J* = 7.4 Hz, H-4), 6.62 (1H, dd, *J* = 7.5, 7.7 Hz, H-5), 6.56 (1H, d, *J* = 7.8 Hz, H-7), 5.16 (1H, s, H-2), 4.42 (1H, dd, *J* = 5.1, 2.4 Hz, H-12), 3.57 (1H, dd, *J* = 11.9, 4.2 Hz, H-9), 3.46 (1H, dd, *J* = 14.3, 2.1 Hz, H-15a), 3.21 (1H, dd, *J* = 14.3, 5.2 Hz, H-15b), 2.86 (3H, s, H-16), 1.89 (1H, dd, *J* = 11.8, 4.5 Hz, H-8α), 1.55 (1H, dd, *J* = 11.9, 12.0 Hz, H-8β); ^1H NMR (600 MHz, $CDCl_3$) symmetrical: δ 7.57 (2H, dd, *J* = 7.5, 7.3 Hz, H-19/21), 7.52 (1H, dd, *J* = 7.5, 7.5 Hz, H-20), 7.15 (2H, d, *J* = 7.2 Hz, H-18/22), 7.09 (1H, dd, *J* = 7.5, 7.3 Hz, H-6), 6.99 (1H, d, *J* = 7.2 Hz, H-4), 6.72 (1H, dd, *J* = 7.3, 7.3 Hz, H-5), 6.57 (1H, d, *J* = 7.6 Hz, H-7), 4.84 (1H, s, H-2), 4.28 (1H, dd, *J* = 4.3, 2.8 Hz, H-12), 3.68 (1H, dd, *J* = 11.9, 4.1 Hz, H-9), 3.54 (1H, dd, *J* = 14.3, 2.9 Hz, H-15a), 3.27 (1H, dd, *J* = 14.3, 4.3 Hz, H-15b), 3.04 (3H, s, H-16), 2.03 (1H, dd, *J* = 12.3, 4.3 Hz, H-8α), 1.59 (1H, dd, *J* = 11.9, 12.2 Hz, H-8β). ^{13}C NMR ($CDCl_3$, 150 MHz) symmetrical: δ 165.4 (C, C-10), 164.0 (C, C-13), 150.2 (C, C-7a), 134.5 (C, C-17), 129.6

(CH, C-6), 129.4 (2× CH, C-18/22), 129.3 (2× CH, C-19/21), 126.5 (C, C-3a), 125.7 (CH, C-4), 118.9 (CH, C-5), 109.6 (CH, C-7), 78.7 (CH, C-2), 63.1 (CH, C-12), 58.9 (C, C-3), 58.6 (CH, C-9), 36.2 (CH_2, C-8), 36.0 (CH_2, C-15), 32.6 (CH_3, C-16). For the X-ray crystal structure, see Figure S2.

Aflatoxin B1 (**10**): White powder; $[\alpha]_D^{20}$ −163° (*c* 3.6, $CHCl_3$); UV ($CHCl_3$) λ_{max} (logε) 240 (3.84), 265 (3.81), 361 (3.94) nm; ESI-MS *m/z* 313.1 $[M + H]^+$; ^{1}H NMR (600 MHz, DMSO-d_6) δ 6.93 (1H, d, *J* = 6.5 Hz, H-9), 6.73 (1H, s, H-7), 6.71 (1H, dd, *J* = 2.0, 2.2 Hz, H-10), 5.38 (1H, dd, *J* = 2.3, 2.4 Hz, H-11), 4.76 (1H, d, *J* = 6.4 Hz, H-12), 3.91 (3H, s, H-17), 3.27 (2H, t, *J* = 5.2 Hz, H-2), 2.47 (2H, overlap, H-3). ^{13}C NMR (150 MHz, DMSO-d_6) δ 201.5 (C, C-1), 178.0 (C, C-15), 165.4 (C, C-4), 161.8 (C, C-8), 154.8 (C, C-6), 152.5 (C, C-14), 146.1 (CH, C-10), 116.9 (C, C-16), 113.9 (CH, C-11), 107.6 (C, C-13), 103.8 (C, C-5), 102.8 (CH, C-7), 91.8 (CH, C-9), 57.5 (CH_3, C-17), 47.4 (CH, C-12), 35.3 (CH_2, C-2), 29.2 (CH_3, C-3).

7-*O*-Acetylkojic acid (**11**): Yellow powder; UV (MeOH) λ_{max} (logε) 208 (3.23), 251 (2.97), 263 (3.85) nm; ESI-MS *m/z* 185.0 $[M + H]^+$; ^{1}H NMR (600 MHz, DMSO-d_6) *δ* 8.09 (1H, s, H-3), 6.47 (1H, s, H-6), 4.94 (2H, s, H-7), 2.11 (3H, s, CH_3). ^{13}C NMR (150 MHz, DMSO-d_6) δ 174.3 (C, C-1) 170.3 (C, Ac), 162.1 (C, C-2), 146.5 (C, C-4), 140.4 (CH, C-6), 113.0 (CH, C-3), 61.7 (CH_2, C-7), 20.8 (CH_3, Ac).

Kojic acid (**12**): Yellow clear crystal. For the X-ray crystal structure, see Figure S4.

*Preparation of MTPA esters of **3a** and **3b***. Compound **3** (450 μg for each) was reacted with either *R*- or *S*-MTPA-Cl (20 μL) in 250 μL of pyridine for 2 h. The reaction mixture was diluted with H_2O and extracted with EtOAc three times. The organic layers were combined and separated by HPLC (70% MeOH in H_2O) to afford the *S*- or *R*-MTPA esters, **3a** and **3b**. *S*-MTPA ester (**3a**): ^{1}H NMR (600 MHz, DMSO-d_6) δ 6.15 (s, 1H, H-4), 6.62 (d, 1H, *J* = 2.1 Hz, H-5), 6.43 (d, 1H, *J* = 2.2 Hz, H-7), 2.74 (dd, 1H, *J* = 14.8, 7.9 Hz, H-9a), 2.83 (dd, 1H, *J* = 14.9, 4.6 Hz, H-9b), 5.74 (m, 1H, H-10), 3.03 (d, 2H, *J* = 6.3 Hz, H-11), 2.14 (s, 3H, H-13), 3.88 (s, 6H, H-14/15); ESI-MS *m/z* 366.2 $[M + H]^+$. *R*-MTPA ester (**3b**): ^{1}H NMR (600 MHz, DMSO-d_6) δ 6.45 (s, 1H, H-4), 6.62 (d, 1H, *J* = 2.0 Hz, H-5), 6.59 (d, 1H, *J* = 2.1 Hz, H-7), 2.84 (dd, 1H, *J* = 14.8, 8.0 Hz, H-9a), 2.91 (dd, 1H, *J* = 14.7, 4.5 Hz, H-9b), 5.75 (m, 1H, H-10), 2.96 (d, 2H, *J* = 6.3 Hz, H-11), 2.05 (s, 3H, H-13), 3.88 (s, 3H, H-14), 3.88 (s, 3H, H-15); ESI-MS *m/z* 366.2 $[M + H]^+$.

Chemical Transformation of leporin B (6) into leporin A (6a). Compound **6** (8 mg, 0.022 mmol) was added to the solution of NaOMe (100 mg, 2*N* in MeOH) and MeI (2 mL). The mixture was heated at 45 °C for 2 h. The solvent was removed under reduced pressure, and the residue was dissolved in EtOAc (10 mL) and washed with H_2O (3 × 10 mL). The organic layer was dried over anhydrous Na_2SO_4 and then was concentrated under reduced pressure. The obtained gum was separated by HPLC (80% MeOH/H_2O) to afford leporin A (**6a**, 3.2 mg, 40% yield), whose structure was identified by a comparison of ^{13}C NMR, specific rotation and MS with those reported [6].

X-ray Crystal data for 9 (Cu-Kα radiation). Colorless orthorhombic crystal (1:1 MeOH-CH_2Cl_2), $C_{42}H_{44}N_6O_6$, space group *P*2(1)2(1)2(1) with *a* = 12.7957 (3) Å, *b* = 14.6222 (3) Å, *c* = 19.5466 (5) Å, *V* = 3657.19 (15) Å^3, *Z* = 4, D_{calcd} = 1.324 mg/m^3, μ = 0.729 mm^{-1}, and *F* (000) = 1544. Crystal size: 0.42 × 0.30 × 0.27 mm^3. Independent reflections: 6,362 with R_{int} = 0.0407. Absolute structure

parameter: 0.2(2). The structure was solved by the direct method (SHELXS-97) and refined using the SHELXL-97 method. The final agreement factors are R1 = 0.0505 and wR2 = 0.1392 ($I > 2\sigma(I)$). Data were obtained on a Bruker Smart CCD area detector diffractometer with graphite monochromated Cu-Kα radiation (λ = 1.54178 Å). Crystallographic data for **9** (Cu-Kα) has been deposited in the Cambridge Crystallographic Data Centre as supplementary publication No. CCDC986948. The data can be obtained free of charge from the Cambridge Crystallographic Data Centre.

X-ray Crystal data for 12 (Mo-Kα radiation). Yellow orthorhombic crystal (MeOH), $C_6H_6O_4$, space group $P2(1)2(1)2(1)$ with a = 3.8337 (4) Å, b = 18.4165 (18) Å, c = 8.5088 (12) Å, V = 596.93 (12) Å^3, Z = 4, D_{calcd} = 1.581 mg/m^3, μ = 0.136 mm^{-1}, and F (000) = 296. Crystal size: 0.50 × 0.43 × 0.21 mm^3. Independent reflections: 1056 with R_{int} = 0.0638. The structure was solved by the direct method (SHELXS-97) and refined using the SHELXL-97 method. The final agreement factors are R1 = 0.0405 and wR2 = 0.1004 ($I > 2\sigma(I)$). Data were obtained on a Bruker Smart CCD area detector diffractometer with graphite monochromated Mo-Kα radiation (λ = 0.71073 Å). Crystallographic data for **12** (Mo-Kα) has been deposited in the Cambridge Crystallographic Data Centre as supplementary publication No. CCDC986947. The data can be obtained free of charge from the Cambridge Crystallographic Data Centre.

Theory and Calculation Details. The calculations were performed by using the density functional theory (DFT) as carried out in Gaussian 03 [7,8]. The preliminary conformational distributions search was performed by HyperChem 7.5 software. All ground-state geometries were optimized at the B3LYP/6-31G(d) level. Solvent effects of methanol solution were evaluated at the same DFT level by using the SCRF/PCM method [9]. TDDFT at B3LYP/6-31G(d) was employed to calculate the electronic excitation energies and rotational strengths in methanol [10]. The stable conformations obtained at the B3LYP/6-31G(d) level were further used in magnetic shielding constants at the B3LYP/6-311++G(2d,p) level and OR computations at the B3LYP/6-311G(d,p) level, respectively.

Table S1. ^{1}H (600 MHz) and ^{13}C NMR (150 MHz) Data for Compounds **1**, **2**, **4** and **5** in DMSO-d_6 (TMS, δ ppm).

Position	1		2		4		5	
	δ_C	δ_H (J in Hz)	δ_C	δ_H (J in Hz)	δ_C	δ_H (J in Hz)	δ_C	δ_H (J in Hz)
1	-	-	-	-	-	-	-	-
2	87.7, CH	4.42, s	86.4, CH	4.03, s	87.4, CH	4.41, s	87.3, CH	4.41, s
3	195.9, C	-	195.6, C	-	197.4, C	-	197.4, C	-
4	118.7, CH	5.71, s	116.4, CH	5.81, s	117.2, CH	5.73, s	117.2, CH	5.74 s
4a	173.2, C	-	155.0, C	-	170.2, C	-	170.2, C	-
4b	40.5, CH	3.12, ddd (11.2, 2.9, 2.9)	74.3, C	-	76.5, C	-	76.4, C	-
5	23.8, CH_2	α 1.87, m; β 1.41, m	32.3, CH_2	α 1.95, m; β 1.85, m	32.1, CH_2	α 2.00, m; β 1.81, m	32.3, CH_2	α 2.02, m; β 1.83, m
6	24.1, CH_2	α 1.73, m; β 1.66, m	21.6, CH_2	α 1.93, m; β 1.67, m	21.2, CH_2	α 1.89, m; β 1.62, m	21.4, CH_2	α 1.93, m; β 1.64, m
6a	48.5, CH	2.67, m	49.7, CH	2.71, m	48.3, CH	2.63, m	48.9, CH	2.63, m
7	27.6, CH_2	α 2.59, dd (6.3, 13.0); β 2.28, dd (10.5, 13.0)	27.4, CH_2	α 2.61, dd (12.8, 6.2); β 2.32 dd (12.8,11.0)	26.4, CH_2	α 2.48, m; β 1.98, m	26.4, CH_2	α 2.47, m; β 1.97, m
7a	116.3, C	-	115.4, C	-	114.7, C	-	115.3, C	-
7b	124.7, C	-	124.8, C	-	123.5, C	-	125.1, C	-
8	114.9, CH	7.20, d (1.5)	114.9, CH	7.20, d (8.5)	116.0, CH	6.85, s	118.1, CH	7.28, d (7.2)
9	139.0, C	-	138.7, C	-	141.3, C	-	118.9, CH	6.91, dd (7.0, 7.6)
10	119.6, CH	6.96, dd (8.5, 1.7)	118.6, CH	6.95, dd (1.9, 8.5)	119.3, CH	6.87, d (7.1)	119.7, CH	6.94, dd (7.8, 7.3)
11	111.9, CH	7.22, d (8.5)	111.8, CH	7.21, d (1.8)	111.3, CH	7.20, d (7.2)	112.4, CH	7.27, d (7.3)
11a	138.7, C	-	138.7, C	-	139.2, C	-	140.6, C	-
12	-	10.52, s	-	10.62, s	-	10.64, s	-	10.60 s
12a	150.9, C	-	153.4, C	-	152.5, C	-	153.3, C	-
12b	51.2, C	-	50.6, C	-	50.6, C	-	51.4, C	-
12c	37.3, C	-	43.3, C	-	40.5, C	-	40.6, C	-
13	29.2, CH_2	α 2.40, m; β 1.92, m	31.0, CH_2	α 3.01, dd (17.1, 2.3); β 2.40, dd (17.3, 6.2)	34.1, CH_2	α 2.75, m; β 2.43, m	34.1, CH_2	α 2.74, m; β 2.32, m
14	28.3, CH_2	α 2.22, m; β 1.81, m	111.4, CH	5.64, m	28.4, CH_2	α 2.72, m; β 1.87, m	28.4, CH_2	α 2.70, m; β 1.89, m
14a	104.2, C	-	145.6, C	-	104.7, C	-	104.7, C	-
12b-Me	15.3, CH_3	1.06, s	16.9, CH_3	1.29, s	16.6, CH_3	1.31, s	16.8, CH_3	1.32, s
12c-Me	21.6, CH_3	1.03, s	20.2, CH_3	1.00, s	22.9, CH_3	1.12, s	23.0, CH_3	1.12, s
1′	77.6, C	-	73.4, C	-	78.4, C	-	78.4, C	-

Table S1. *Cont.*

Position	1		2		4		5	
	**δ_C**	**δ_H (J in Hz)**	**δ_C**	**δ_H (J in Hz)**	**δ_C**	**δ_H (J in Hz)**	**δ_C**	**δ_H (J in Hz)**
2′	29.1, CH_3	1.367, s	27.6, CH_3	1.25, s	29.0, CH_3	1.38, s	29.0, CH_3	1.38, s
3′	23.5, CH_3	1.11, s	27.2, CH_3	1.14, s	23.4, CH_3	1.09, s	23.4, CH_3	1.09, s
1″	41.0, C	-	41.2, C	-	41.5, C	-	-	-
2″	149.5, CH	6.04, dd (10.6, 17.4)	149.5, CH	6.04, dd (10.6, 17.4)	149.7, CH	6.23, dd (10.6, 17.3)	-	-
3″	110.3, CH_2	4.99, dd (1.5, 10.6); 5.02, dd (1.5, 17.4)	110.3, CH_2	5.03, dd (17.5, 1.5); 5.00, dd (10.6, 1.5)	111.8, CH_2	4.98, d (10.5); 4.84, d (17.3)	-	-
4″	29.1, CH_3	1.37, s	29.1, CH_3	1.38, (s)	29.9, CH_3	1.45, s	-	-
5″	29.5, CH_3	1.24, s	29.1, CH_3	1.38, (s)	29.7, CH_3	1.44, s	-	-
4b-OH	-	-	-	4.91, s	-	5.06, s	-	5.08, s
1′-OH	-	-	-	4.55, s	-	-	-	-

Table S2. The measured and calculated ^{13}C NMR Data for **1** and (2*S*,14a*R*)-**1**.

Position	Measured 1	Calcd. 1			Calcd. (2*S*,14a*R*)-1		
	δ_C	δ_C	Corrected 1	Error	δ_C	Corrected (2S,14a*R*)-1	Error
1	-	-	-	-	-	-	-
2	87.7	90.8	82.0	5.7	71.4	64.7	23.0
3	195.9	209.1	187.7	8.2	188	172.8	23.1
4	118.7	134.4	120.9	−2.2	131.4	120.3	−1.6
4a	173.2	207.4	186.2	−13.0	194.4	178.7	−5.5
4b	40.5	48	43.7	−3.2	49.3	44.2	−3.7
5	23.8	26.8	24.8	−1.0	30	26.3	−2.6
6	24.1	27.7	25.6	−1.5	27.8	24.3	−0.2
6a	48.5	53.7	48.8	−0.3	54.4	49.0	−0.5
7	27.6	30	27.6	0.0	29.4	25.8	1.8
7a	116.3	130.6	117.5	−1.2	129.4	118.5	−2.2
7b	124.7	141.6	127.4	−2.7	140.9	129.1	−4.4
8	114.9	122.6	110.4	4.5	123.1	112.6	2.3
9	139.0	157.9	142.0	−3.0	158.4	145.4	−6.4
10	119.6	127.5	114.8	4.8	127.2	116.4	3.2
11	111.9	125.5	113.0	−1.1	125	114.4	−2.5
11a	138.7	155.7	140.0	−1.2	155.4	142.6	−3.8
12a	150.9	160.9	144.6	6.3	160.4	147.2	3.7
12b	51.2	57.8	52.5	−1.2	55.8	50.2	1.0
12c	37.3	44.5	40.6	−3.3	49.6	44.5	−7.2
13	29.2	31.6	29.0	0.1	35.4	31.3	−2.2
14	28.3	32	29.4	−1.2	33.1	29.2	−1.0
14a	104.2	110.9	99.9	4.2	102.3	93.4	10.8
12c-Me	21.6	23.8	22.1	−0.5	16.2	13.5	8.0
12b-Me	15.3	14.7	13.9	1.3	15.4	12.8	2.5
1'	77.6	80.2	72.5	5.1	107.4	98.1	−20.5
2'	29.1	30.6	28.1	1.0	27.5	24.0	5.1
3'	23.5	24.5	22.7	0.8	40.9	36.4	−13.0
1''	41.0	46.1	42.0	−1.0	45.9	41.1	−0.1
2''	149.5	169.1	152.0	−2.5	169.1	155.3	−5.8
3''	110.3	120.8	108.8	1.5	120.9	110.6	−0.3
4''	29.1	31.4	28.9	0.2	34.6	30.6	−1.5
5''	29.5	34.8	31.9	−2.4	31.4	27.6	1.9

Figure S3. The structures of Compounds **1** and (2*S*,14a*R*)-**1**.

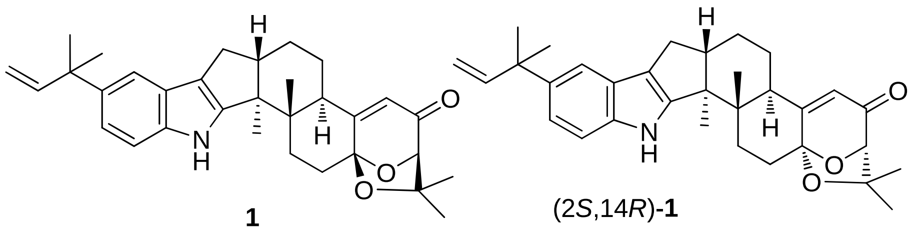

1 (2*S*,14*R*)-**1**

Table S3. Cytotoxicities of Compounds **1–10** against the MCF-7 and A549 cell lines (IC_{50} μM).

**Cell Lines**	**1**	**2**	**3**	**4**	**5**	**6**	**7**	**8**	**9**	**10**
MCF-7	20.3	24.1	66.8	29.4	26.0	57.2	37.6	30.2	78.4	48.1
A549	18.4	22.0	>100	25.7	24.3	46.2	41.2	32.9	>100	43.3

Figure S4. ORTEP drawings of **9** (left, Cu-Kα) and **12** (right, Mo-Kα).

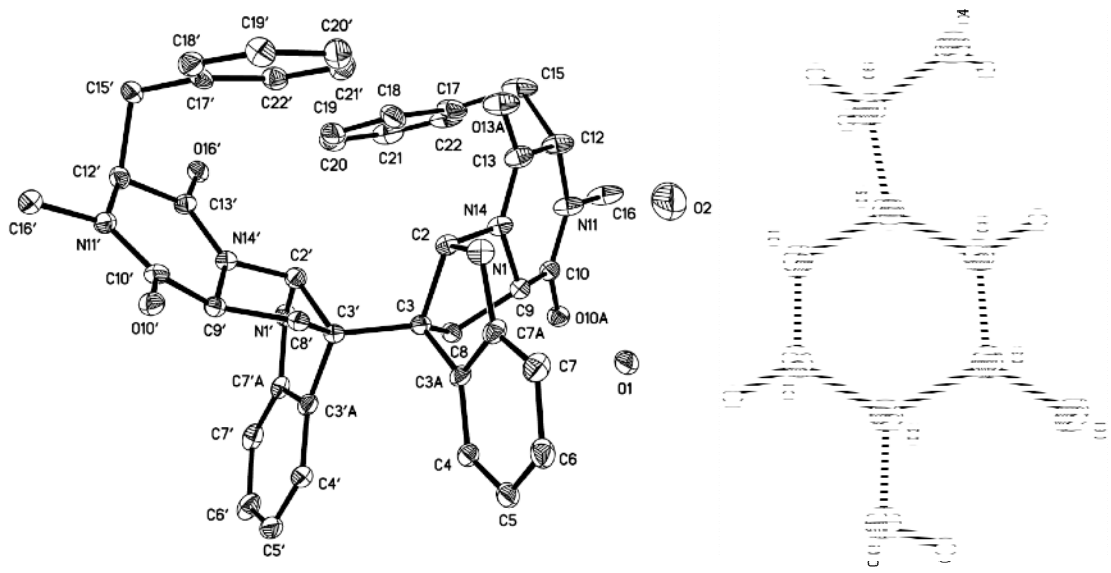

Figure S5. Measured and calculated ECD spectra for **2** and (2*S*)-**2**.

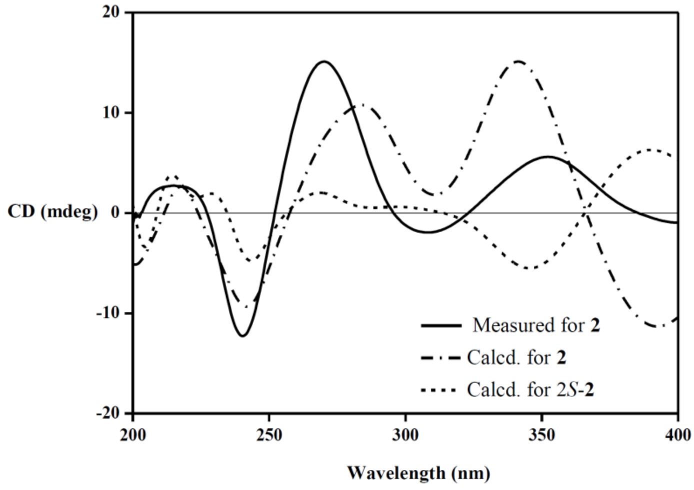

Figure S6. Measured and calculated ECD spectra for **6** and ent-**6**.

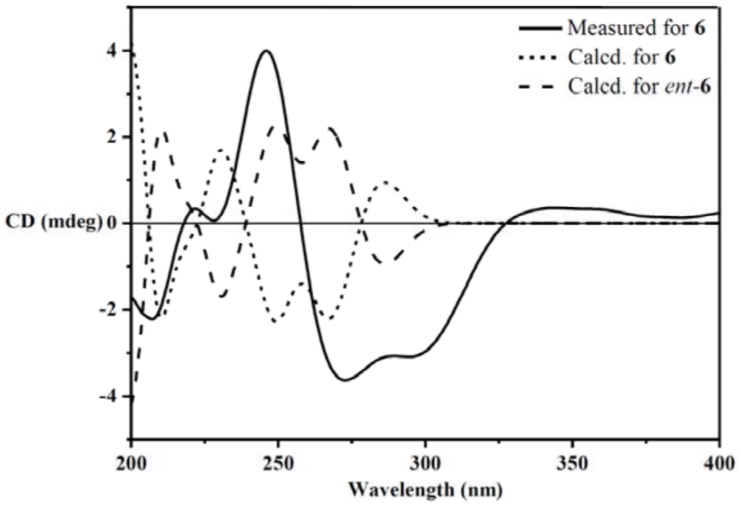

Figure S7. The ^{1}H NMR spectrum of **1** in DMSO-d_6.

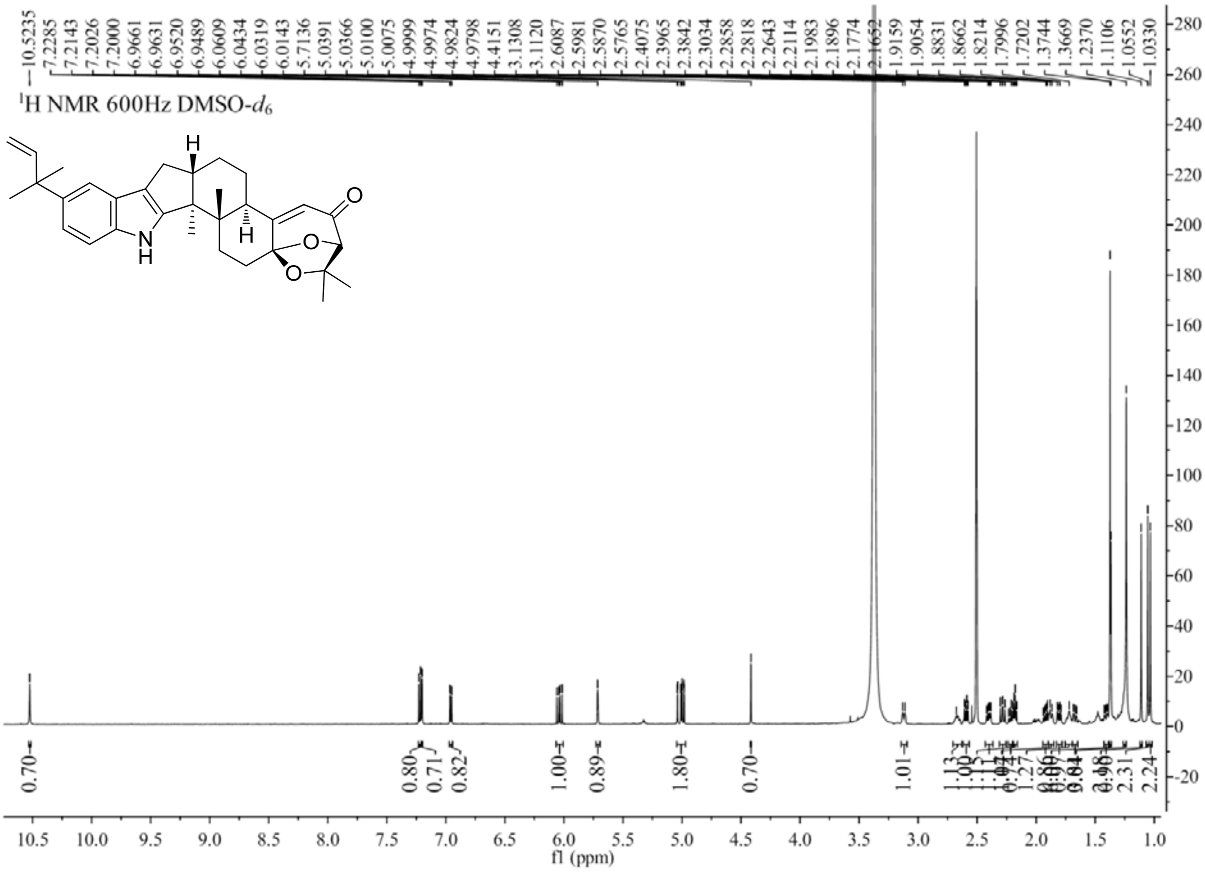

Figure S8. Partial enlarged ^{1}H NMR spectrum of **1** in DMSO-d_6.

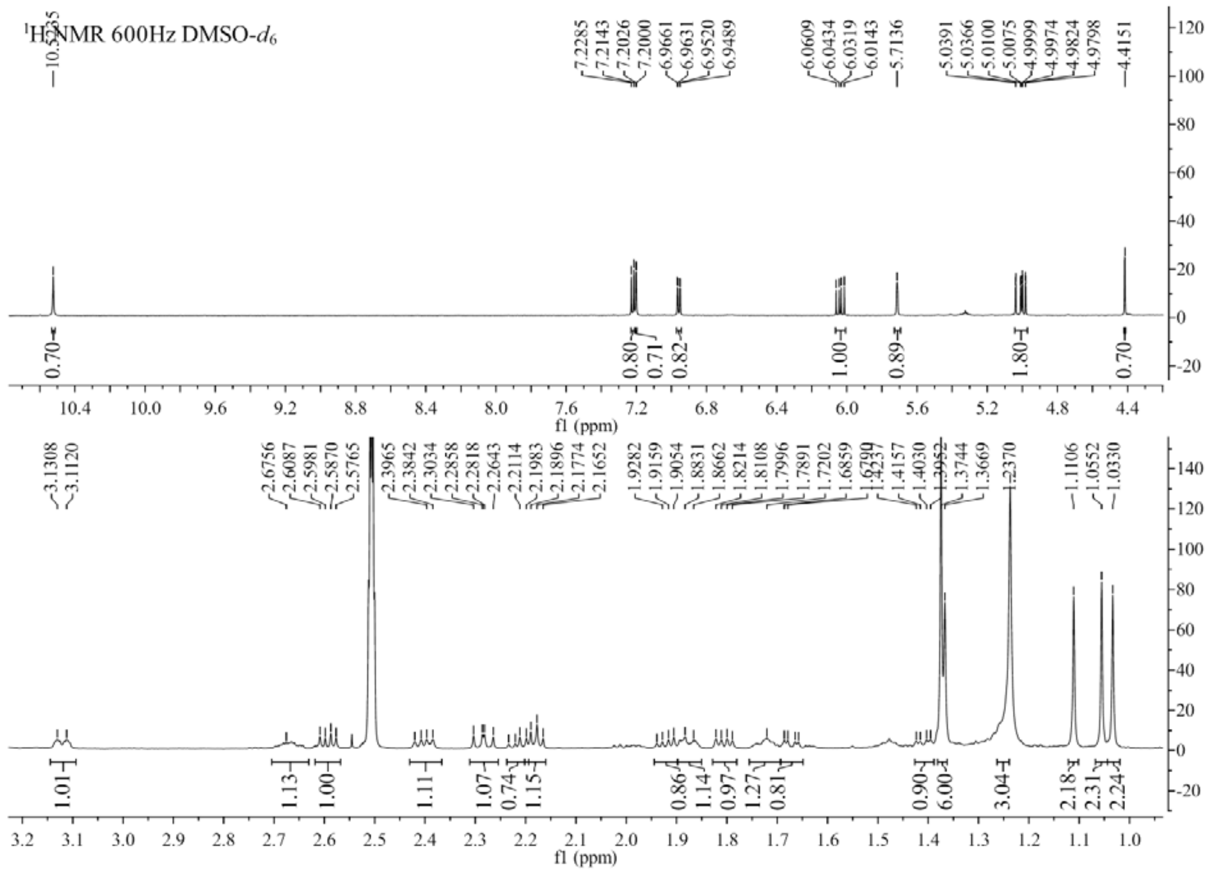

Figure S9. The ^{13}C DEPTQ spectrum of **1** in DMSO-d_6.

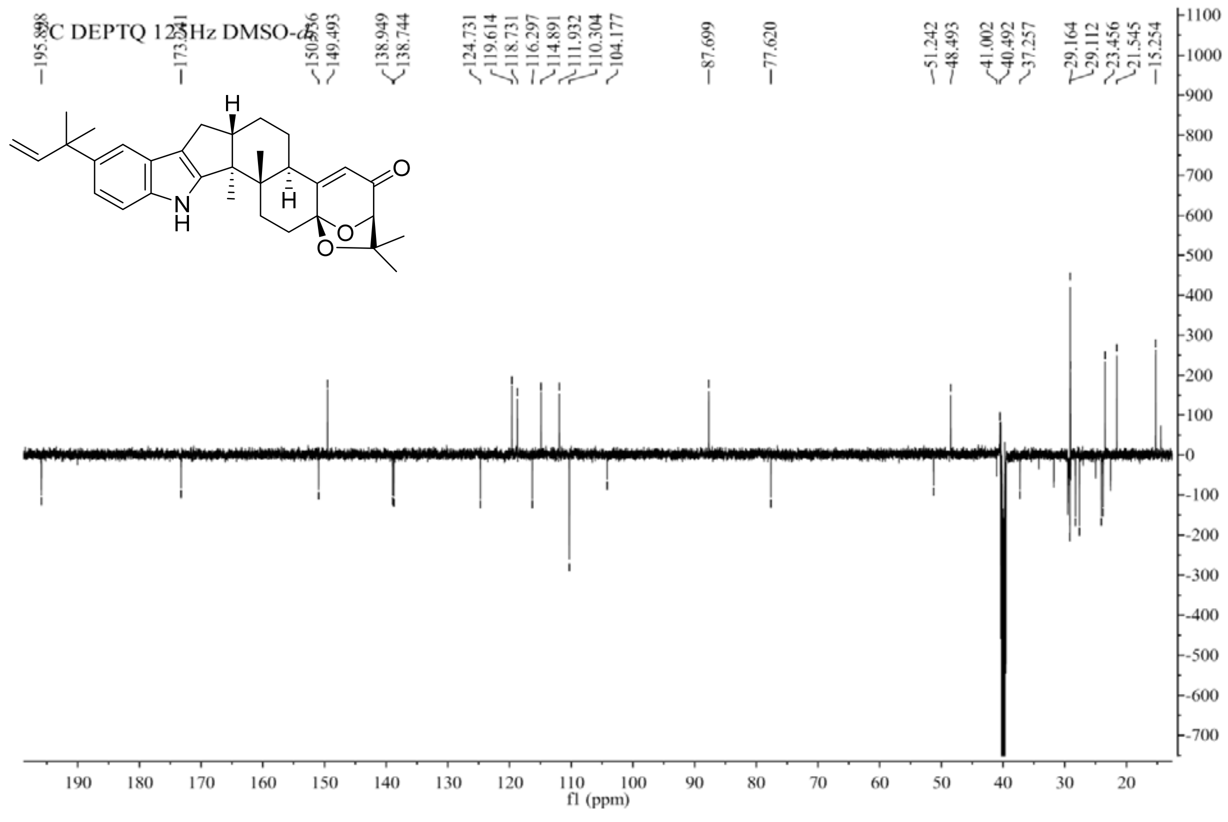

Figure S10. The DEPT spectrum of **1** in DMSO-d_6.

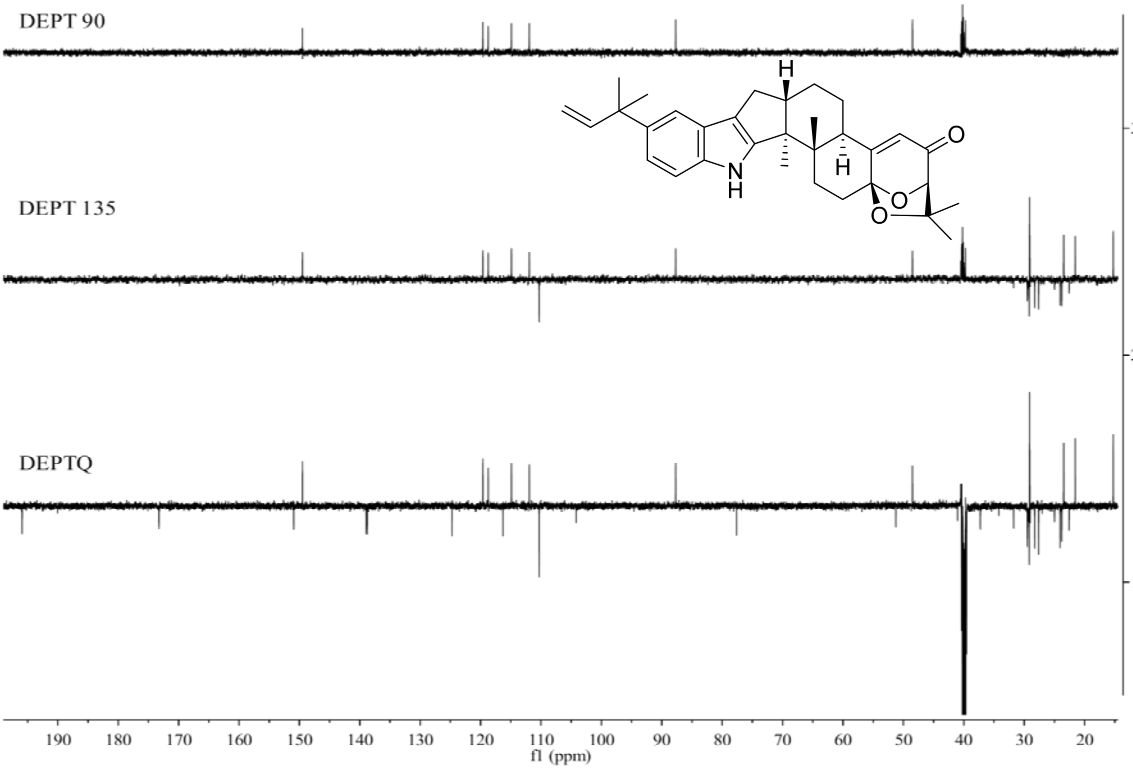

Figure S11. The HSQC spectrum of **1** in DMSO-d_6.

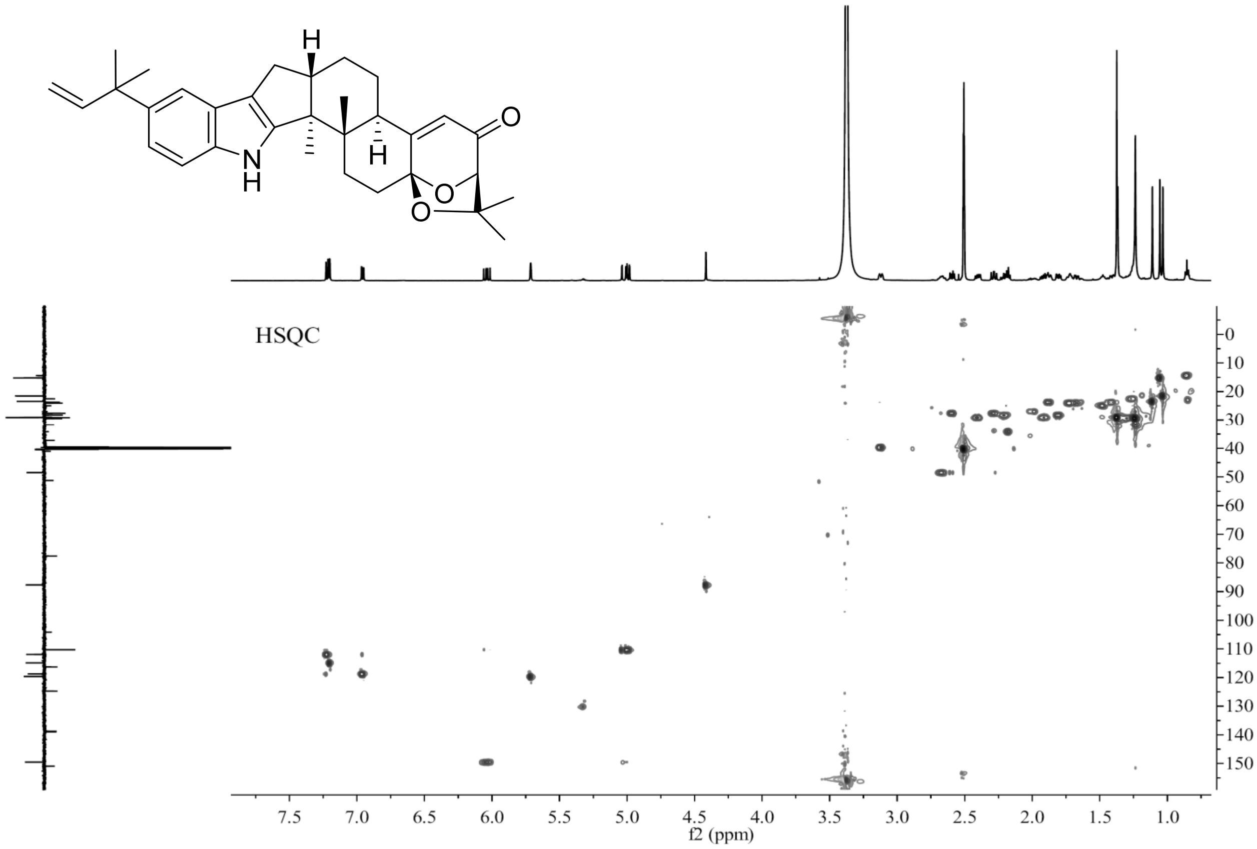

Figure S12. The ^{1}H-^{1}H COSY spectrum of **1** in DMSO-d$_6$.

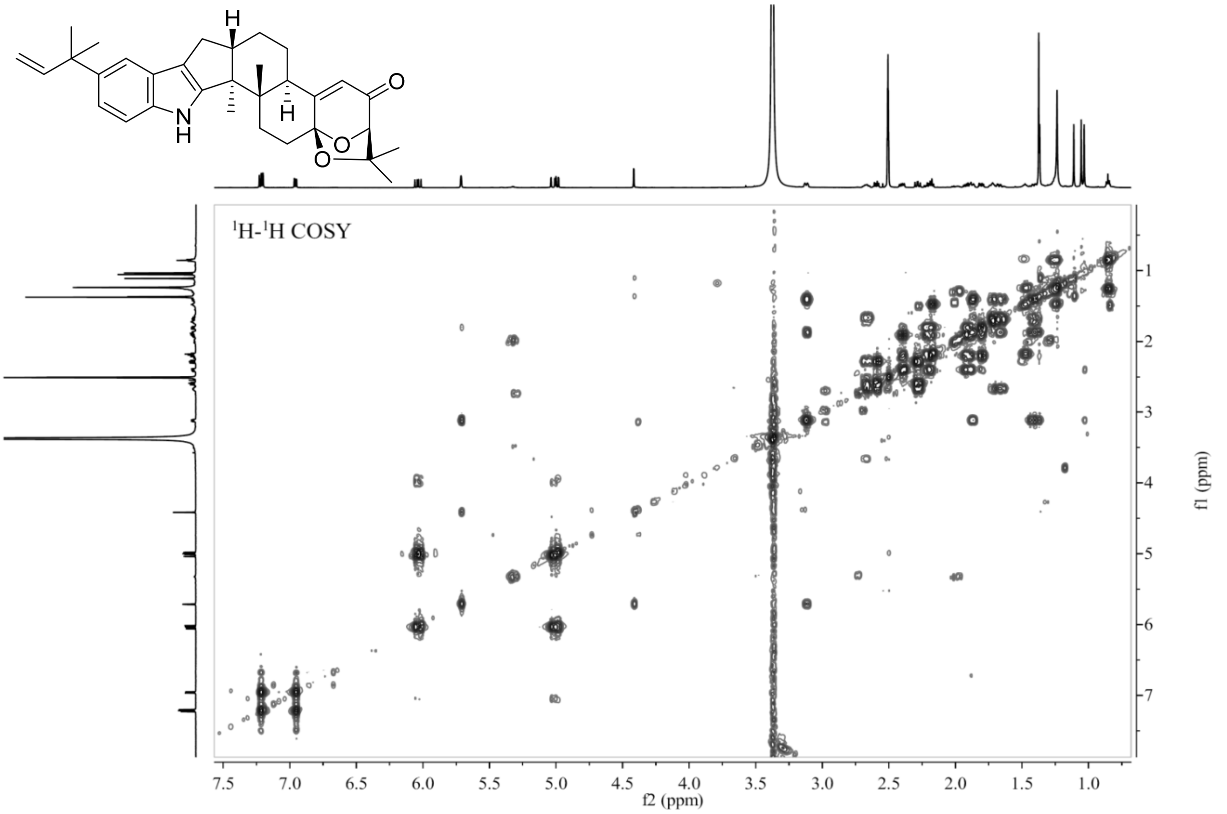

Figure S13. The HMBC spectrum of **1** in DMSO-*d*$_6$.

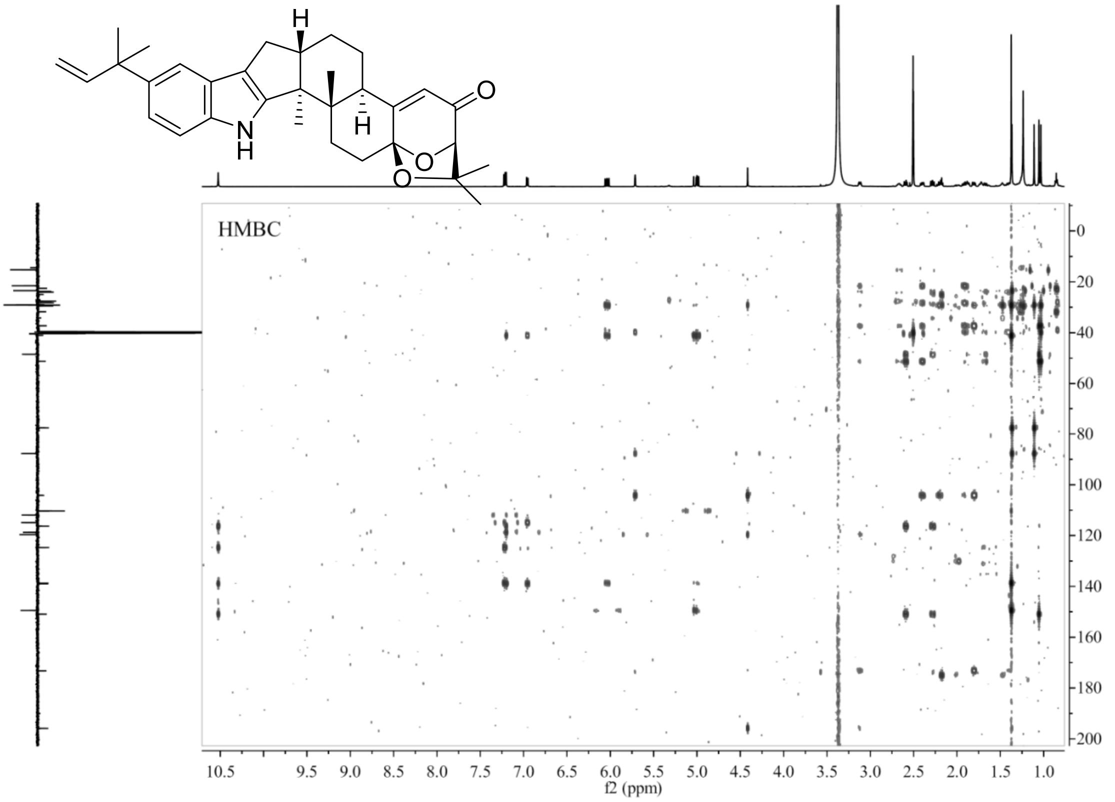

Figure S14. Partial enlarged HMBC spectrum of **1** in DMSO-d_6.

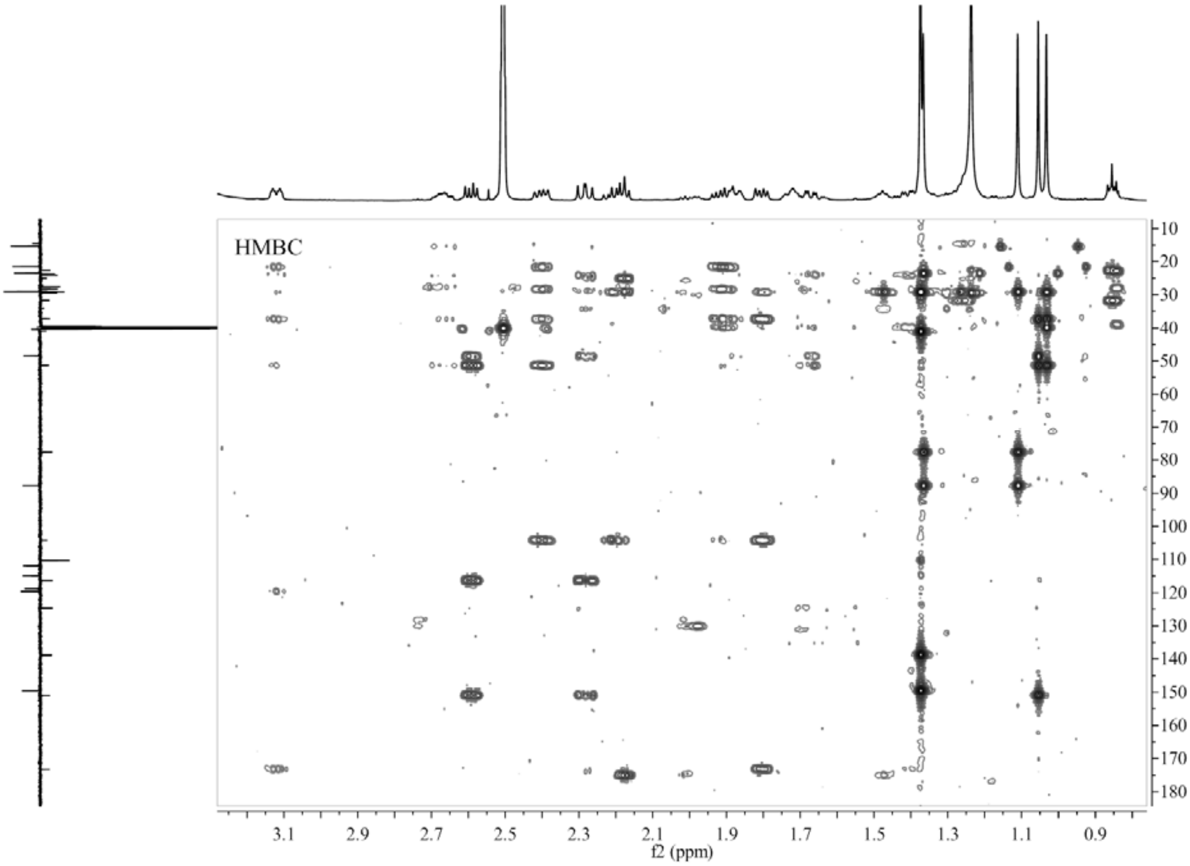

Figure S15. The NOESY spectrum of **1** in DMSO-d_6.

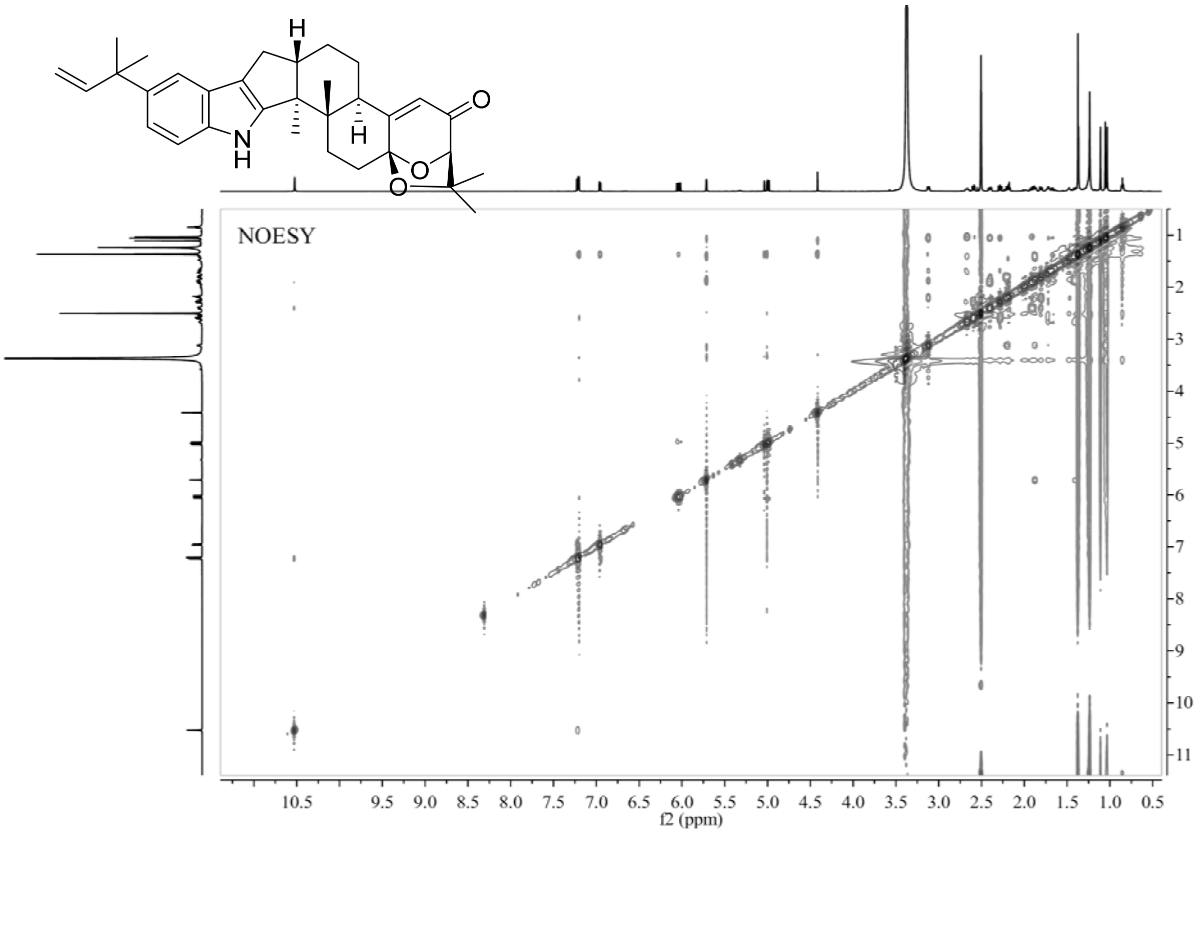

Figure S16. The ^{1}H-NMR spectrum of **2** in DMSO-d_6.

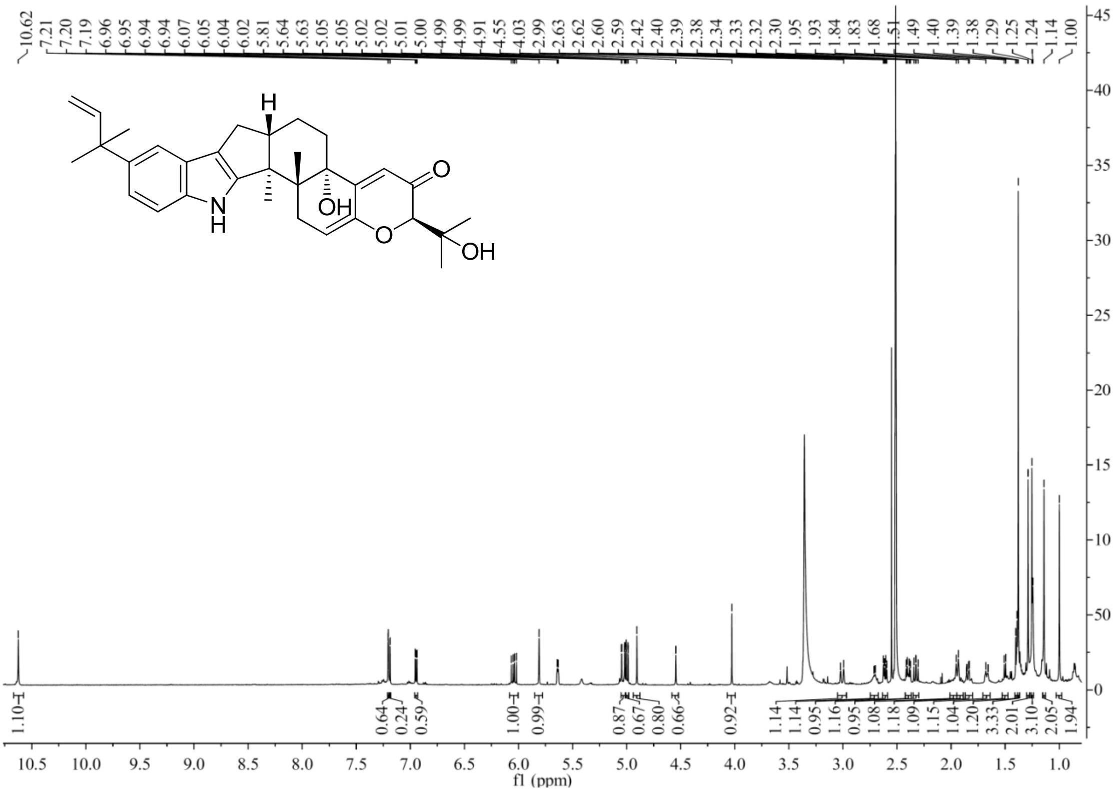

Figure S17. The DEPTQ spectrum of **2** in DMSO-d6.

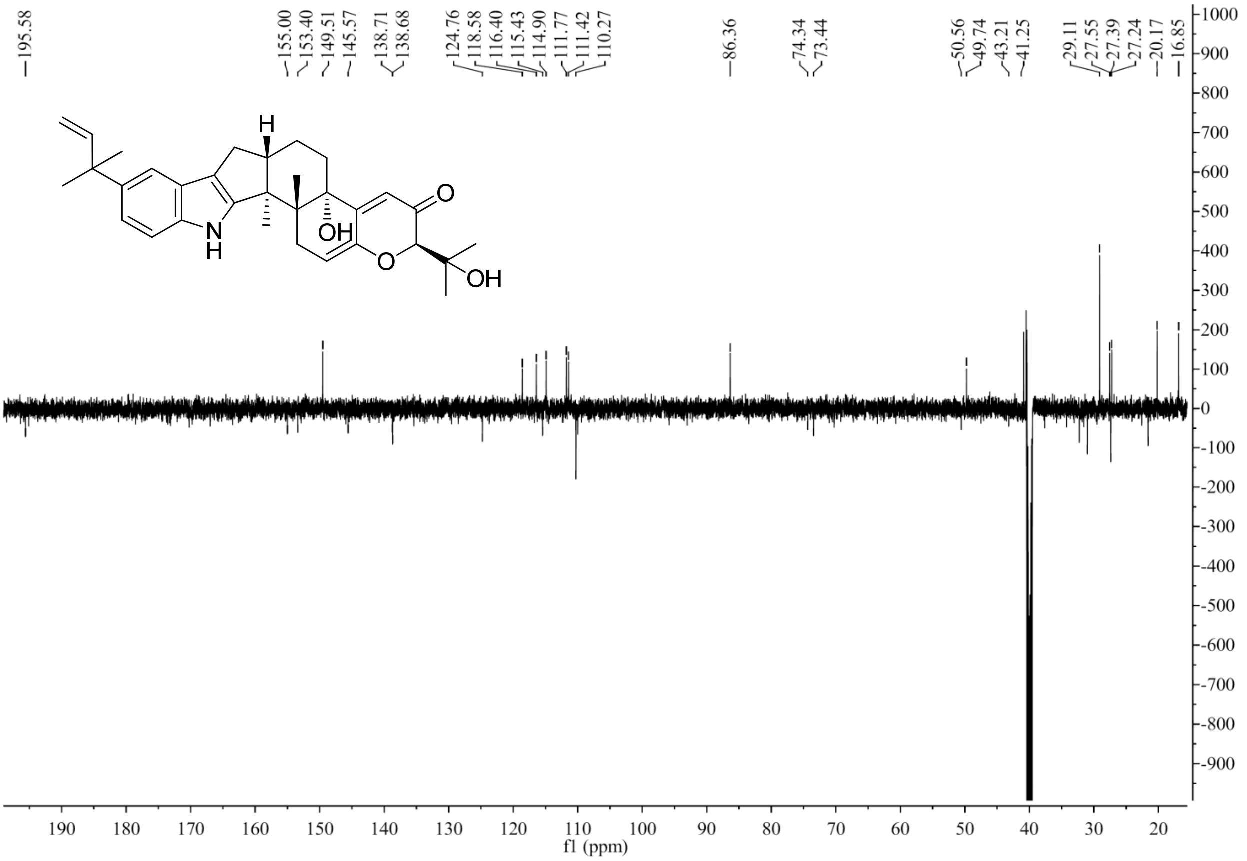

Figure S18. The HMQC spectrum of **2** in DMSO-d_6.

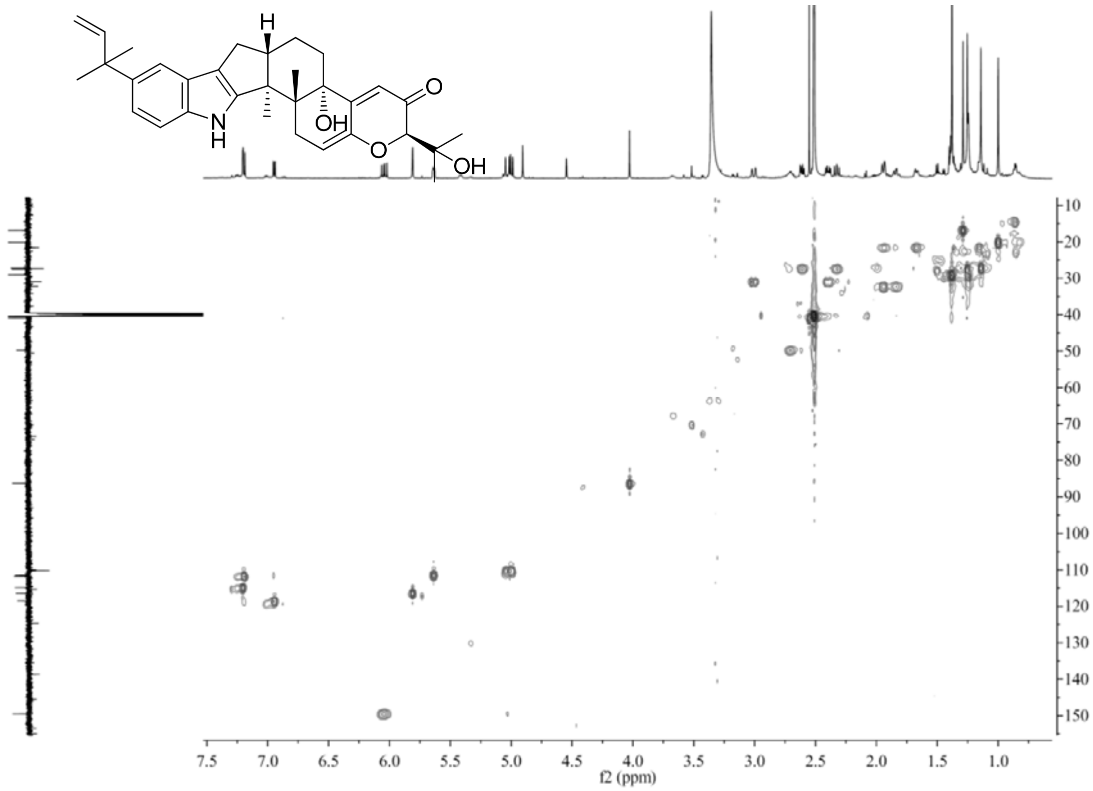

Figure S19. The ^{1}H-^{1}H COSY spectrum of **2** in DMSO-d_6.

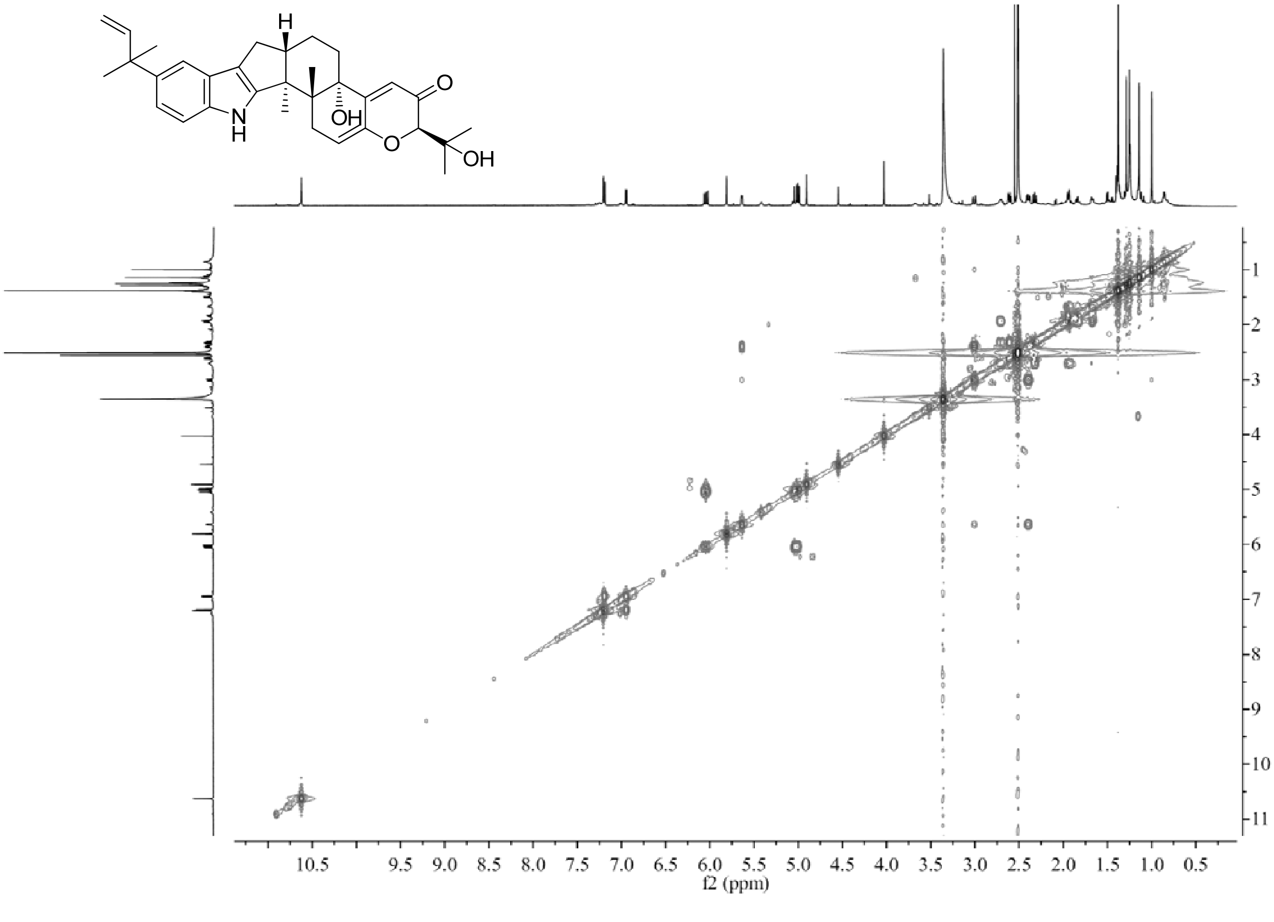

Figure S20. The HMBC spectrum of **2** in DMSO-d_6.

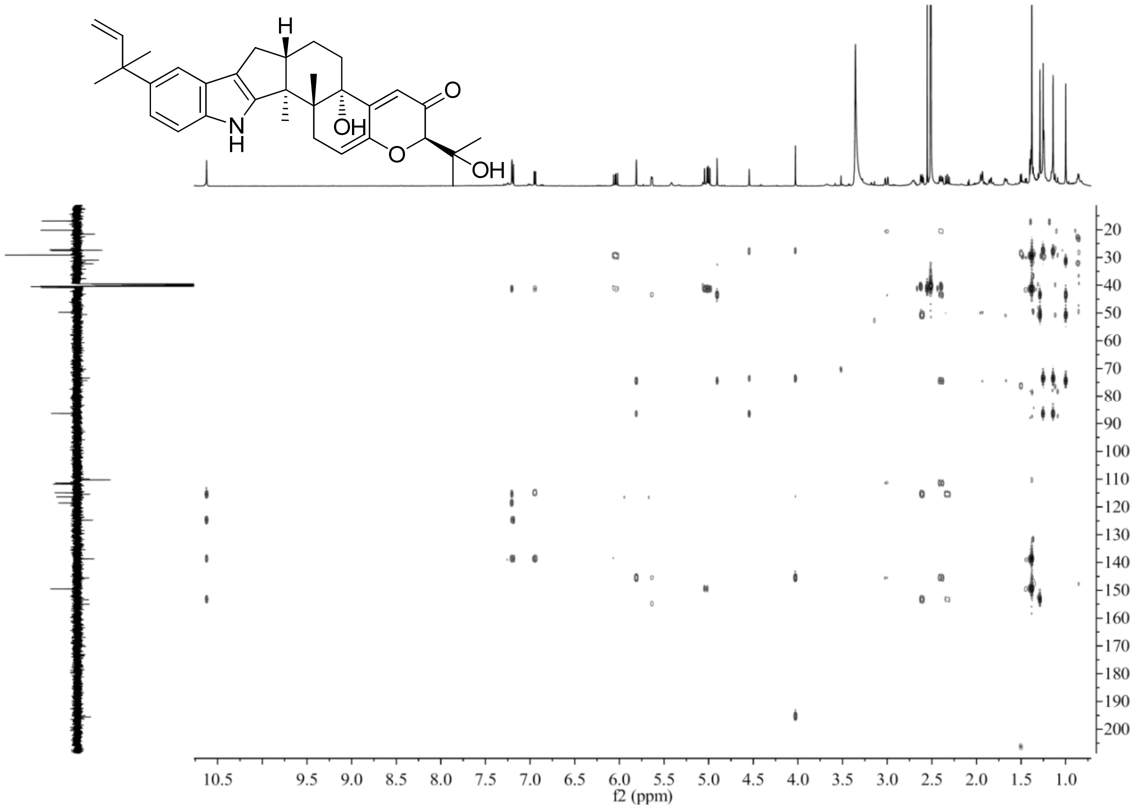

Figure S21. The NOESY spectrum of **2** in DMSO-d_6.

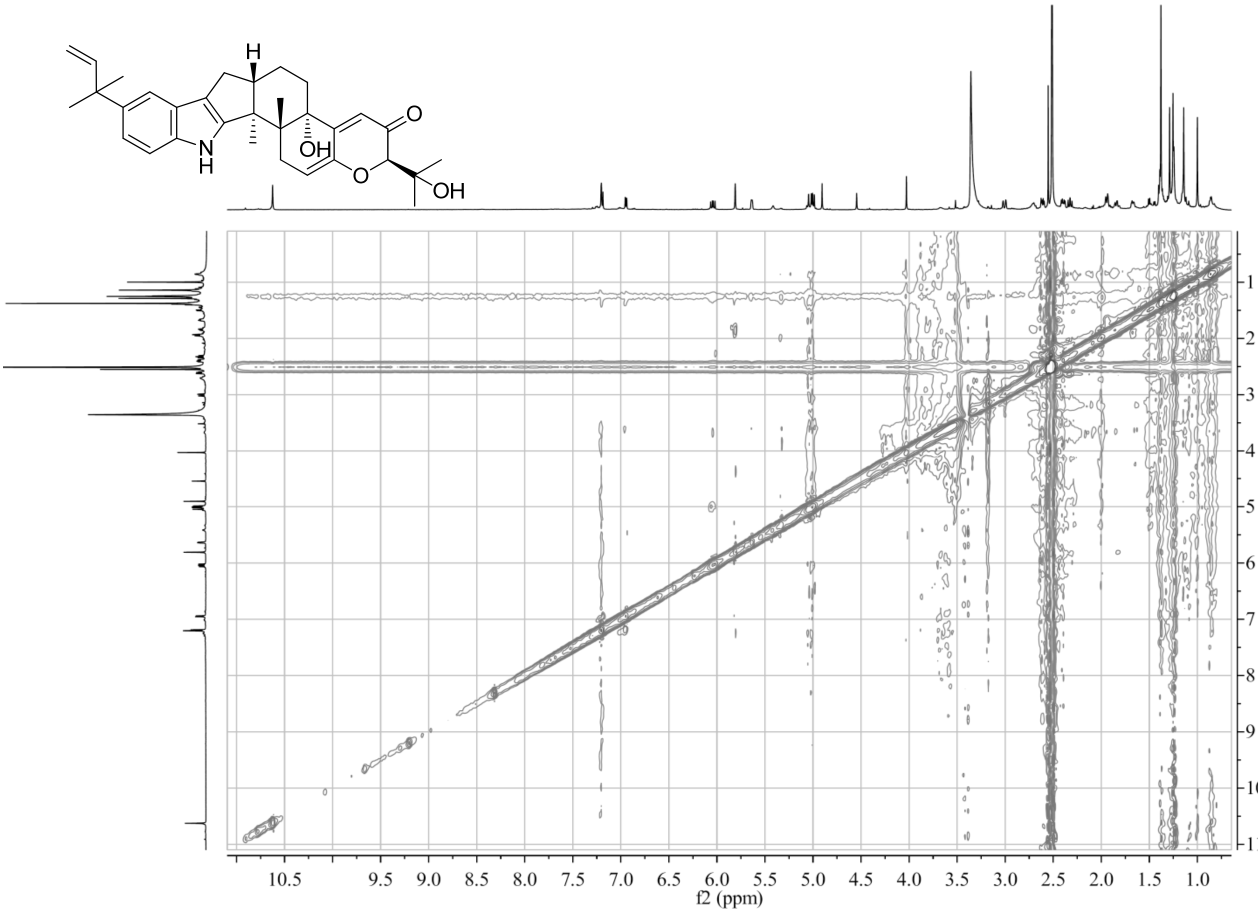

Figure S22. The ^{1}H NMR spectrum of **3** in DMSO-d_6.

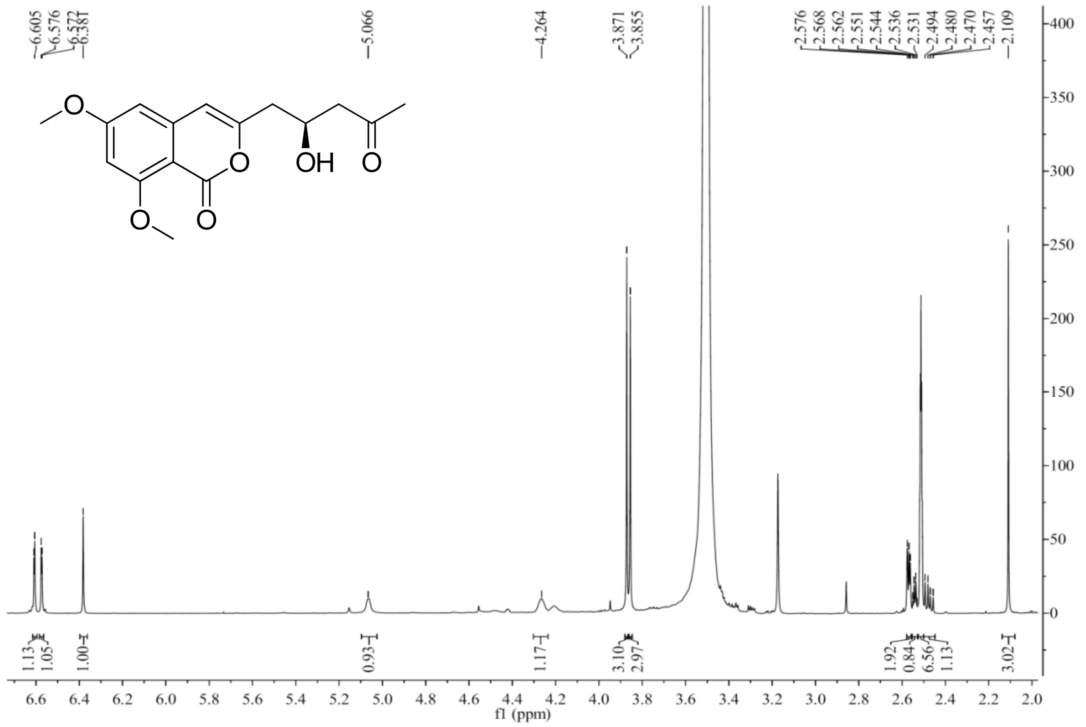

Figure S23. The ^{13}C NMR spectrum of **3** in DMSO-d_6.

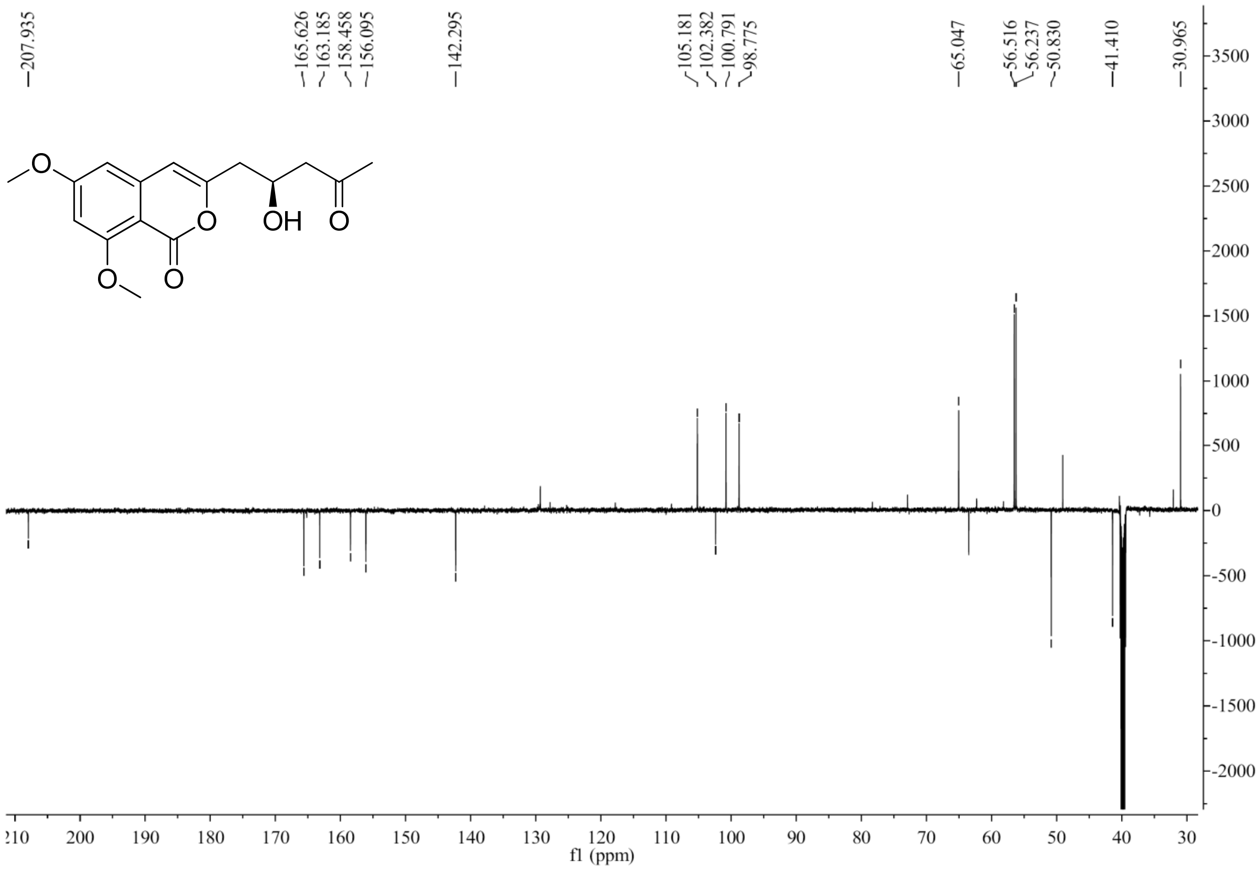

Figure S24. The HMQC spectrum of **3** in DMSO-d_6.

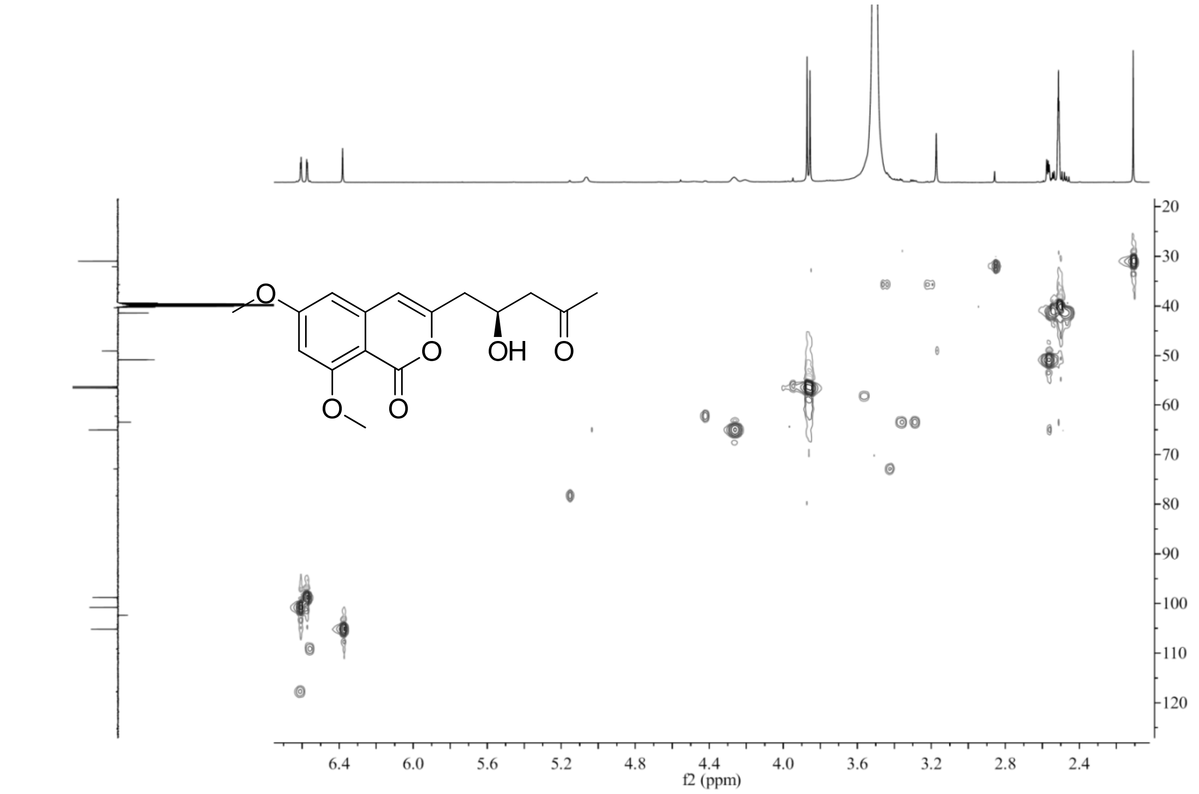

Figure S25. The ^{1}H-^{1}H COSY spectrum of **3** in DMSO-d_6.

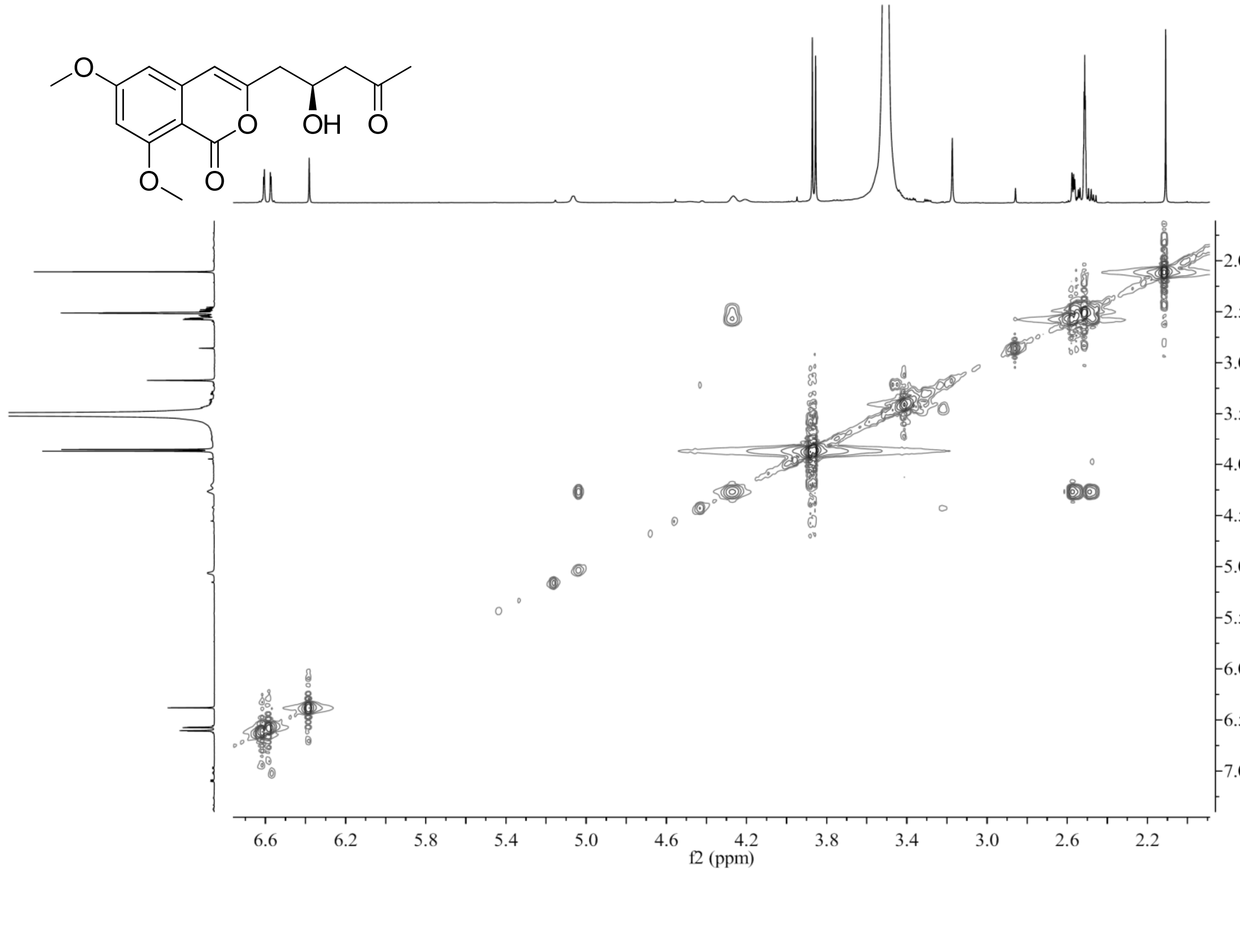

Figure S26. The HMBC spectrum of **3** in DMSO-d_6.

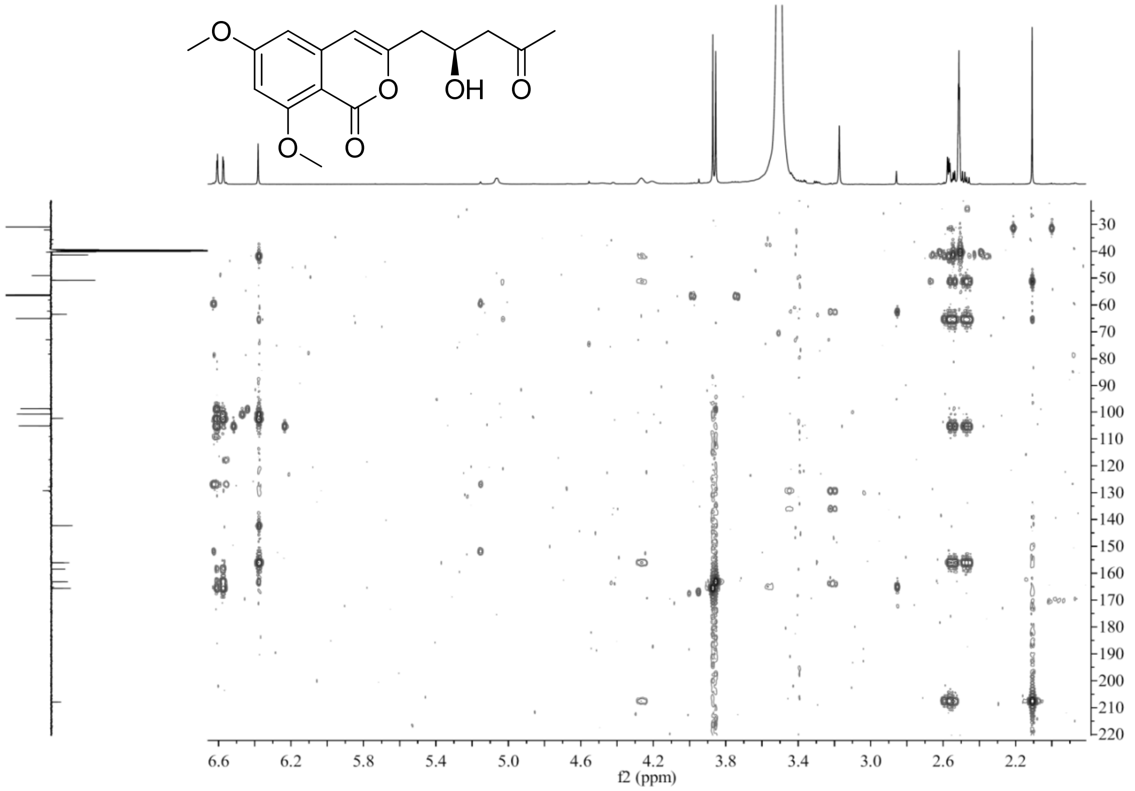

Figure S27. The NOESY spectrum of **3** in DMSO-d_6.

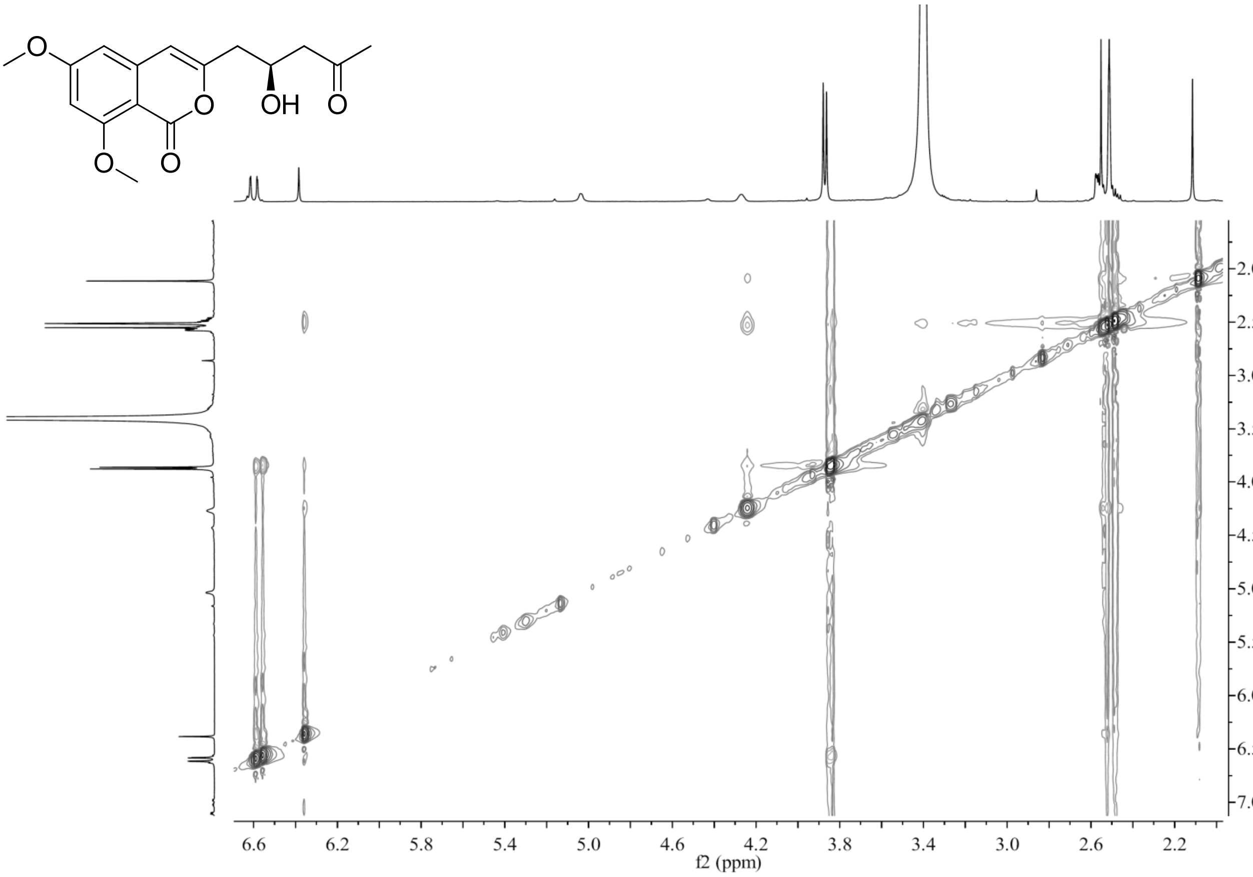

Figure S28. The DNA histograms of A549 cells treated with Compounds **1** and **2** at 10 µM.

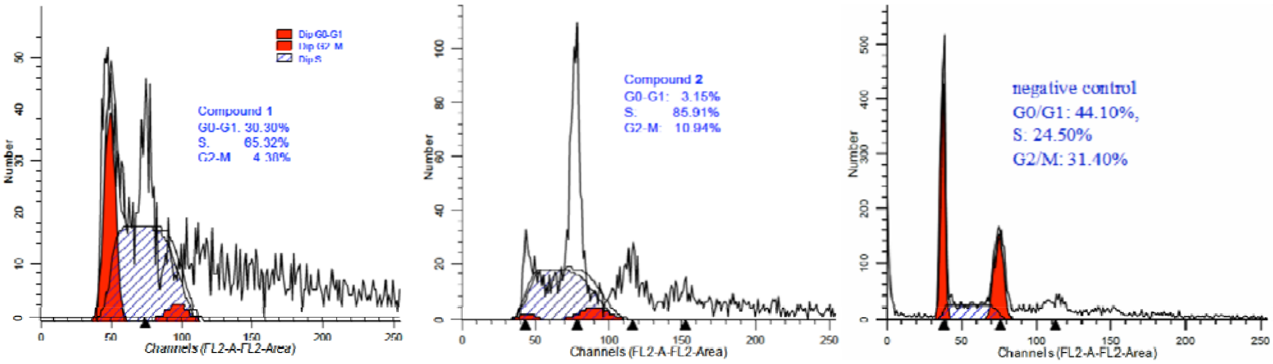

References

1. Mosmann, T. Rapid colorimetric assay for cellular growth and survival: Application to proliferation and cytotoxicity assays. *J. Immunol. Methods* **1983**, *65*, 55–63.
2. Zaika, L.L. Spices and herbs: Their antimicrobial activity and its determination. *J. Food Saf.* **1988**, *9*, 97–118.
3. Li, J.S.; Kleeff, J.; Guo, J.C.; Fischer, L.; Giese, N.; Büchler, M.W.; Friess, H. Effects of STI571 (gleevec) on pancreatic cancer cell growth. *Mol. Cancer* **2003**, *2*, 32.
4. Somberg, R.; Pferdehirt, B.; Kupcho, K. A universal kinase aassay for a world of kinases. *Promega Notes* **2003**, *83*, 14–17.
5. Grassauer, A.; Weinmuellner, R.; Meier, C.; Pretsch, A.; Prieschl-Grassauer, E.; Unger, H. Iota-Carrageenan is a potent inhibitor of rhinovirus infection. *Virol. J.* **2008**, *5*, 107.
6. Tepaske, M.R.; Gloer, J.B. Leporin A: An antiinsectan N-alkoxypyridone from the sclerotia of *Aspergillus leporis*. *Tetrahedron Lett.* **1991**, *32*, 5687–5690.
7. Zheng, J. K.; Zhu, H.J.; Hong, K.; Wang, Y.; Liu, P.P.; Wang, X.; Peng, X. P.; Zhu, W.M. Novel cyclic hexapeptides from marine-derived fungus, Aspergillus sclerotiorum PT06-1. *Org. Lett.* **2009**, *11*, 5262–5265.
8. Frisch, G.W.; Trucks, H.B.; Schlegel, G.E.; Scuseria, M.A.; Robb, J.R.; Cheeseman, J.A.; Montgomery, T., Jr.; Vreven, K.N.; Kudin, J.C.; Burant, J M.; *et al. Pople, Gaussian 03, Revision E. 01, Suite of Programs for ab initio Calculation*; Gaussian, Inc.: Wallingford, CT, USA, 2004.
9. Cammi, R.; Tomasi, J. Remarks on the use of the apparent surface charges (ASC) methods in solvation problems: Iterative versus matrix-inversion procedures and the renormalization of the apparent charges. *J. Comput. Chem.* **1995**, *16*, 1449–1458.
10. Gross, E.K.U.; Dobson, J.F.; Petersilka, M. Density functional theory of time-dependent phenomena. *Density Funct. Theory II* **1996**, *181*, 81–172.

© 2014 by the authors; licensee MDPI, Basel, Switzerland. This article is an open access article distributed under the terms and conditions of the Creative Commons Attribution license (http://creativecommons.org/licenses/by/3.0/).
